# Perceived inequality and variability in the expression of parochial altruism

**DOI:** 10.1017/ehs.2024.43

**Published:** 2025-01-20

**Authors:** Cody T. Ross, Anne C. Pisor

**Affiliations:** 1 Max Planck Institute for Evolutionary Anthropology, Department of Human Behavior, Ecology and Culture, Leipzig, Germany; 2 Department of Anthropology and Social Science Research Institute, The Pennyslvania State University, University Park, PA, USA; 3 Department of Anthropology, Washington State University, Pullman, WA, USA

**Keywords:** Parochial altruism, parochialism, inter-ethnic cooperation, inter-ethnic conflict, sociality

## Abstract

It is commonly argued that humans have generalised predispositions for within-group favouritism and between-group animus (i.e. that humans are *parochially altruistic*), leading to higher levels of internal conflict in societies with greater diversity. Other research, however, has questioned both the ubiquity of parochial altruism and the role of diversity *per se* in causing social discord. Here, we use ethnographic, social network and experimental economic game data to explore this topic in two multi-ethnic Colombian communities. We examine the extent to which Afrocolombian and Emberá residents express parochial altruism, finding appreciable variability between communities, and across individuals within communities. When present, parochial altruism appears to be driven by divergent perceptions of group-based economic need, not group identity *per se*. Our results suggest that diversity may be less likely to cause social discord than past work has suggested, as long as group-based inequalities in wealth, well-being and representation – that can destabilise positive inter-group relationships – are minimised.

## Graphical abstract

**Figure:**
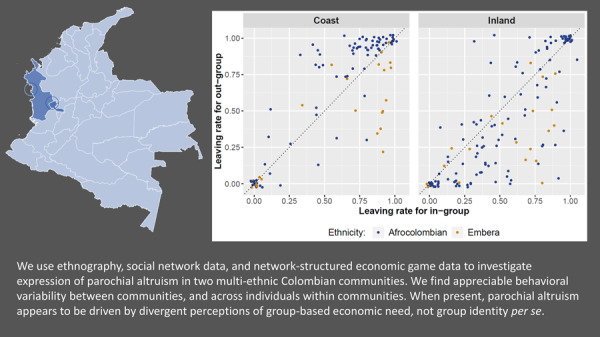

**Social media summary:** Perceived inequality affects expression of parochial altruism in Afrocolombian and Emberá communities in rural Colombia.

## 1. Introduction

Humans sometimes behave in a *parochially altruistic* manner (Bowles, 2008) – that is, they engage in within-group cooperation coupled with some degree of out-group hostility or animus (see Supporting Information, SI, Section 1.1 for notes on terminology). However, the intensity and prevalence of such parochial altruism appear to vary, sometimes substantially, across human groups and across contexts (de Dreu et al., 2014; Böhm et al., 2020). While some of this variation is probably due to variability in the methods used to study the phenomena (Pisor & Ross, 2023), variation in the socio-ecological context of inter-group relations seems to have profound effects on inter-personal behaviour. At times, markers of identity – e.g. language, ethnic background, or religion – come to delineate hard boundaries that structure behaviour, while in other cases potential markers of identity do not lead to pronounced differences in how people treat one another. The relationship between identity group (see SI, Section 1.2), the structuring of cooperation and conflict, and resultant socio-cultural outcomes thus remains a central focus of study across the social sciences – from anthropology to political science and economics.

Controversial work in the 1990s and 2000s found identity-group diversity (specifically ethnic diversity) to be negatively associated with a variety of socio-cultural outcomes serving as proxies of ‘the public good’ (e.g. see Easterly & Levine, 1997; Alesina et al., 1999; Alesina & Ferrara, 2005). These arguments posit that where identity-group diversity is high, individuals of a particular group will cooperate amongst themselves, while competing with individuals of other groups, in turn leading to less efficient systems at some higher level of organisation (like the county, or nation). These studies, however, have been critiqued, with researchers arguing that diversity *per se* may not have negative impacts on higher-level socio-cultural outcomes, but rather that perceptions of zero-sumness between groups and structures of group-based dominance (e.g. discriminatory legal frameworks, apartheid systems, and unjust power or resource imbalances) – that *sometimes* arise in the presence of diversity – lead to such outcomes (e.g. Waring & Bell, 2013; Wimmer et al., 2009; Beheim & Bell, 2024). In other words, identity-group diversity itself may pose little threat to wide-scale cooperation if institutions ensuring just treatment of diverse ethnic or other identity groups are maintained. Empirical work has since explored the effects of ethnically-structured inequality on higher-order socio-cultural outcomes (e.g. Alesina et al., 2016), finding evidence that between-group inequality may indeed play a mediating role in the correlation between ethnic diversity and adverse socio-cultural outcomes uncovered in earlier work.

Most empirical test of these ideas, however, do not directly assess the underlying causal model by measuring both: (i) whether cooperation *and* animus are structured by group-identity in real-world social networks; and (ii) whether inter-group relations are causal in the resulting socio-cultural outcomes. Instead, ecological regressions and rather coarse, national or administrative-district data are used to test if aggregate-level indicators of socio-cultural outcomes are associated with aggregate-level metrics of diversity (Pisor & Ross, 2023). Such approaches are subject to unmeasured confounding (Wakefield, 2003) and the ecological inference problem (see Piantadosi et al., 1988; Wakefield, 2004; Lawson et al., 2015; Ross & Winterhalder, 2016).

In this paper, we focus on point (i) – whether cooperation *and* animus are structured by group identity in real-world social networks. We designed a fine-scale, quantitative study of how identity-group structures both positive and negative social ties in a near-complete census of two rural, multi-ethnic, Colombian communities. More specifically, we draw on social network data and network-structured economic games to explore variation in the expression of, and preferences for, parochial altruism in two populations composed of Afrocolombian and Indigenous Emberá individuals. To measure variation in overt behaviour, we draw on network analysis methods to study structure in social and food/money sharing networks (Koster & Leckie, 2014). To measure variation in preferences for cooperation with, exploitation of, and animus towards others, we draw on network-structured variants of classic economic games (see Gervais, 2017). Additionally, we integrate qualitative methods to assess how site-level variables might influence the degree to which parochial altruism is expressed.

As we are interested in how different contexts of inter-ethnic interaction impact expression of parochial altruism, we selected two study sites in Colombia – one on the ethnic boundary between Afrocolombian and Emberá groups, and one in a territory where Afrocolombians have demographic prominence. The sites differ in key ways, especially in the perceptions of between group wealth inequality, as we describe in more detail later on. To guide our interpretation of statistical differences between sites, we draw on ethnographic observations and qualitative interviews with respondents (Geertz, 1973), gathered over periods of several months living in these communities. In what remains of the paper, we review the literature on inter-group relations and then present our empirical work on perceived between-group wealth inequality and parochial altruism in rural Colombia. We conclude by commenting on the potential value of individual-level, network-based approaches for studying inter-group relations and socio-cultural outcomes more broadly.

### 1.1. Conflict and cooperation between groups

When parochial altruism emerges with particular intensity, it can drive some of the most abhorrent of human behaviours: warfare, apartheid and genocide all stem from behavioural processes in which cooperation – or in a weaker sense, coordination – between in-group members is used to compete with or harm out-group members (see SI, Section 1.3). Such behaviours seem omnipresent in human history: antisemitism against European Jews has been present from the Edict of Expulsion in 1290 to the Holocaust in the 1940s and beyond (Brustein & King, 2004); in the 1840s to the 1870s in California, settlers and government actors engaged in the genocide of Native Americans (Lindsay, 2012; Madley, 2016); in 1904–1908, Germans occupying what is now Namibia did the same to Herero and Namaqua groups (Erichsen & Olusoga, 2010); from the 1940s to the 1990s an Apartheid regime in South Africa institutionalised a brutal system of racial segregation (Clark & Worger, 2013); and, in 1994, in the span of only a few months, Hutu militias in Rwanda killed several hundred thousand members of Tutsi and Twa ethnic groups, nearly a sixth of the country's population (Magnarella, 2005).

Given such a history of between-group conflicts, some scholars have come to view parochial altruism, or at least predispositions towards it, as something of a universal of human psychology, arguing that cognitive mechanisms that lead us to *value* in-group members and *devalue* out-group members have a deep evolutionary history, and are nearly inexorable (Clark et al., 2019). This view, however, has also been challenged for some time (e.g. Cashdan, 2001, see also Sections 5.3 and 5.4).

Cases of inter-group cooperation – although perhaps less studied and more prosaic – seem just as omnipresent in human history (Fearon & Laitin, 1996; Glowacki, 2022). Even in the cases of violence described above, parochialism was not universal among members of the aggressing groups – and cosmopolitan altruism (Galston, 1993) could be found undergirding incredible acts of bravery. Yad Vashem – The World Holocaust Remembrance Center – has officially recognised more than 27,000 individuals from more than 50 countries as rescuers who risked their own lives to protect Jewish individuals from Nazis and fascists (Fogelman, 2011). In Rwanda too, the same heroism was shown by some Hutu, with some rescuers going as far as claiming (Rothbart & Cooley, 2016: 92):

I would lock the house with my family [and a rescued Tutsi child] inside, and I'd stand outside. I would tell them, ‘If you are going to kill her, then go ahead and burn the entire house, throw a grenade and kill all of them! They are all my children! If you are going to kill her, then kill me too!’

and many moderate Hutu were themselves murdered by other Hutu precisely because they risked everything to defend out-group members (Rothbart & Cooley, 2016). Many white South Africans rose in resistance to racist policies, and nearly a third of the anti-apartheid activists tried for high treason in the 1956 Treason Trial were of white or Indian background (Shimoni, 1988). More than 100 years after the genocide in Namibia, representatives in Germany, hearing the calls from the Herero people, began to work towards formally accepting historic and moral responsibility for their nation's actions (Sarkin & Fowler, 2008), and pledged 1.1 billion Euros towards aid programmes in Nambia as a gesture of reconciliation (Dagdelen et al., 2021).

### 1.2. Valuation of out-group members

Although inter-group relations are often viewed broadly through the lens of parochial altruism (Böhm et al., 2020), intergroup relations are not always competitive or hostile (Fearon & Laitin, 1996; Brewer, 2010; Jha, 2013). An individual's attitude about an out-group member – that is, the value they place on them (e.g. Gervais & Fessler, 2017) – can be positive, neutral, or negative. In most parts of the world, there are routine interactions between individuals of differing identity groups. Fearon (2003), for example, studied ethnic diversity across 160 countries and found 822 ethnic groups with population sizes reaching at least 1% of their host countries’ total population, and remarks that in places like Papua New Guinea, ethnic diversity is so high that no group is large enough to make up even 1% of the population. Similarly, Eberhard et al. (2021) suggest that there are more than 7,000 languages spoken around the world, and Hunter (2014) argues that there are more than 4,000 religious identity groups. While all of these estimates are sensitive to methodology – especially the lumping vs. splitting of group identities – they highlight the order of human cultural diversity (see also Bell et al., 2009; Richerson et al., 2016). In spite of this diversity, most interactions in most human societies are characterised by tolerance, if not outright cooperation, rather than animus (Fearon & Laitin, 1996); see also Brewer (2010), Böhm et al. (2020) and Riek et al. (2006) for relevant reviews. It is thus important to identify the mechanisms which lead to a breakdown of congenial relationships across identity boundaries, rather than viewing parochial altruism as a *condicio sine qua non*.

In some contexts, especially when population sizes are small, individuals may judge others on the basis of individual-level traits, regardless of identity-group similarity or dissimilarity. However, in larger populations, often characterised by a ‘small-world’ network structure (Watts & Strogatz, 1998) – where tight, homophilic clusters are linked by cross-cluster ties – individuals begin using heuristics to judge anonymous alters on the basis of their social group (Dunbar, 2008). Out-groups may become cognitively represented as single entities, and a valuation – positive, neutral, or negative – may then be extended to all group members (Pietraszewski, 2021). When an out-group is negatively valued, individuals of a focal group may treat out-group members with contempt, hold and spread derogatory stereotypes of them, and even dehumanise them (see Moffett, 2013; Haslam, 2006, for reviews). Negative stereotypes often frame out-group members as competitors who can inflict costs on in-group members (Riek et al., 2006; Brewer, 2010; Böhm et al., 2020). Parochial altruism appears most likely to arise in contexts where individuals generalise negative evaluations across anonymous out-group individuals (see de Dreu et al., 2014, for discussion). However, what social forces might cause negative evaluations of out-group members to be more likely?

### 1.3. Mechanisms of interest

There are at least three widely studied (and often overlapping) families of theoretical models linking diversity, out-group valuations, and resultant socio-cultural outcomes: (i) models based on norm differences and coordination; (ii) models based on between-group resource inequality or other forms of inter-group dominance; and (iii) models based on demographic characteristics and perceptions of ‘out-group threat’.

#### 1.3.1. Norm/preference heterogeneity and miscoordination

In the 1990s, economists focused on understanding local-level variation in the funding of public programmes in the United States came to suspect that ethnic diversity might be associated with reduced contributions to the public good (Alesina et al., 1999). Although the implications of this work have been somewhat controversial, Alesina et al. (1999) were intent on understanding the cause of biased behaviour: their data showed that white Americans decrease investment in public programmes as the demographic prominence of other ethnic groups in a given region increases. In their model, a population of voters can decide on the level of investment in public goods (i.e. a tax rate) and on the types of public goods funded through taxation; as the population becomes more fragmented (in terms of norms/preferences for *types* of public investments), overall public investment by the majority group decreases. However, diversity in group identity will not cause norm disalignment as in the Alesina et al. (1999) model unless norms strongly covary with group-identity empirically.

Recognising that norms – and even group identity itself – are often flexible social constructs subject to change (e.g. during enculturation), anthropologists developed mathematical models to explore inter-ethnic coordination in the more general case where norms are dynamic, but nevertheless impact pay-offs in coordination games (e.g. see McElreath et al., 2003). In this family of models, identity-group diversity is not assumed to imply norm diversity *a priori* as in Alesina et al. (1999); instead, norms are frequency dependent and dynamic, so that if migration rates into an area are low, then variation in group identity does not imply variation in norms (i.e. the norms of the majority group may be adopted by newcomers to the majority area).

For this reason, the model of McElreath et al. (2003) predicts that identity group will only be a salient feature for individuals when the costs to miscoordination are high, where norm variation is high, and where norms are tightly correlated with group identity. In such contexts, in-group biases are expected to emerge, as they minimise the frequency of conflicts resulting from norm disalignment (McElreath et al., 2003; Moya & Boyd, 2015; Moffett, 2013). The McElreath et al. (2003) model, however, is also limited in scope, as it assumes that individuals can be described as having, or deploying, only a single kind of norm in inter-personal interactions. In subsequent work (e.g. Bunce & McElreath, 2017, 2018; Bunce, 2021), many assumptions in McElreath et al. (2003) are relaxed. Most notably, Bunce (2021) explores how cross-cultural competency – the ability to understand and coordinate on multiple norms – allows for mutually beneficial inter-group relationships to be maintained, and miscoordination avoided.

Human groups, even those with very different norms, can benefit from cooperating with one another – especially via the mechanism of commerce/trade (e.g. Pinker, 2011; Jha, 2013) – and so we may seek out such relationships, especially when they offer opportunities for mutual gain relative to parochial exclusion (Bowles & Gintis, 2004). At an individual level, this might involve learning and appreciating different ways of doing things (Bunce, 2021). At a broader level, cultural institutions to protect beneficial inter-ethnic relationships can emerge. Jha (2013), for example, shows that medieval trading ports in South East Asia, despite being more ethnically mixed than other localities, were five times less prone to Hindu–Muslim riots between 1850 and 1950. Jha (2013) attributes this finding to the fact that trade and commerce carried significant benefits for members of both groups, and thus enduring cultural institutions and cosmopolitan norms for valuing and coordinating with one another emerged. Contrasting the empirical work of Jha (2013) with much of the literature on inter-group conflict suggests that it is not simply diversity that impacts socio-cultural outcomes: context, norms, and perceived costs and benefits matter.

#### 1.3.2. Resource constraints, between-group inequality and inter-group dominance

To explain humans’ purported predisposition towards within-group favouritism and between-group animus, many evolutionary explanations focus on the problem of gaining and maintaining resource access, which may have acted as a strong selection pressure over long periods of human history (Choi & Bowles, 2007; Seabright, 2004; Wrangham & Glowacki, 2012). If key resources – like potable water, arable land and productive fisheries – are heterogeneously distributed, then groups controlling resource-dense territories would benefit demographically (i.e. increase in size) if their individual members possessed adaptions to both (i) cooperate with fellow in-group members and (ii) compete with out-group members – either by simply denying out-group members access to resources, or more directly by violently competing with out-groups to expand in-group resource control (Bowles, 2008; Bell & Moya, 2021). The coevolutionary mechanism in which between-group competition stabilises within-group cooperation, however, need not be genetically coded: cultural institutions for regulating within- and between-group behaviour are subject to similar coevolutionary dynamics (Zefferman & Mathew, 2015; Richerson et al., 2016). Similarly, the inequality in resource control that might drive between-group competition must be interpreted broadly. Beyond land, water, or material resources, inequality in social affordances, respect, prestige, political representation or a variety of other sociocultual factors may influence between-group relations (e.g. Fiske et al., 2016; Sidanius & Protto, 2001; Henrich & Gil-White, 2001).

At a proximate level, resource control means power (Fiske et al., 2016): a group with resource control can selectively grant or withhold resource access (Balliet et al., 2017), possibly increasing animus and conflict, especially as power becomes concentrated and used to benefit a restricted class of individuals (Montalvo & Reynal-Querol, 2005). Often, the most powerful group is the largest demographically. That said, resource control and power can lie in the hands of the few if their competitive ability is increased by other factors, like access to weapons (e.g. during the conquest of California; Madley, 2016).

Group living has constituted a critical and enduring part of human evolutionary history, and we should thus expect humans to have adaptations for dealing with key problems associated with group living – especially fairness in resource division (Bøggild & Petersen, 2016). If one sub-group in a given population uses its position of power to extract disproportionate benefit, this has the potential to trigger responses from other parties. Groups with less power can form alliances to improve their competitive ability (Redhead & von Rueden, 2021) – indicating some level of flexibility in how humans perceive and act on group identity. Militant groups, for example, often form alliances in the context of conflicts with powerful and repressive governments, and they find ways to enforce coordination, even if trust is initially low or shared norms absent (Bapat & Bond, 2012). Similar forms of alliances can also function within the political realm – e.g. inter-ethnic alliances in Bolivia led to the rise of the first Indigenous president in South America in 2005, and to the passing of a new Bolivian constitution in 2009 (Fontana, 2014). In sum, there is evidence to suggest that power and resource considerations might be a cause of both parochial behaviour and between-group relationships, depending on context.

Given that: (i) strong imbalances in group power, status or resource control have the potential to attenuate between-group cooperation (Waring & Bell, 2013) and even trigger between-group conflict (Alesina et al., 2016); and (ii) conflict is normally costly for the parties involved, there is scope for selection on norms and institutions that reduce status differentiation and bridge group divides. For example, cultural norms for intermarriage between European royal families appear to have decreased European war frequency by extending kinship networks across group boundaries (Benzell & Cooke, 2021). Similarly, there is scope for inter-group concessions from powerful groups towards disempowered groups in order to minimise conflicts. Classical models in foraging theory, like the tolerated theft model (Winterhalder, 1996), would predict that groups in positions of power should make concessions to outgroups, as long as the marginal benefit of the resources they are conceding to the outgroup exceed their own expected resource defence costs (see also Rusch, 2014). Under such a model, we might expect inter-group tolerance to be common when between-group inequality is low, but parochialism to emerge as between-group inequality becomes more extreme (Alesina et al., 2016).

Other cogent models, however, complicate this explanation. Huber and Mayoral (2019), for example, make the case that one must also account for the structure of within-group inequality as well, arguing that – especially in modern-day nation-state contexts – the intensity of civil conflicts is likely to be highest when within-group inequality is high, as such inequality decreases the opportunity cost to poor group members of fighting, and increases the potential *per capita* spoils of war that can be reaped by the rich elite.

#### 1.3.3. Demographic differences and perceptions of ‘out-group threat’

One of the most important characteristics affecting a group's resource holding potential is its population size relative to other groups, as this predicts its ability to win an altercation with another group (Wrangham & Glowacki, 2012; Turchin & Gavrilets, 2009), even if altercations are not violent (Alesina et al., 1999; Posner, 2004). Because group size confers a hegemonic group power in inter-group interactions, political scientists have found that inter-group relationships tend towards parochialism when demographic processes threaten the predominance of the majority group (Slack & Doyon, 2001; Advani & Reich, 2015). Social psychologists have likewise found that individuals are more likely to apply a negative valuation to out-group members when they are perceived as a ‘realistic threat’ to power (see Riek et al., 2006; Brewer, 2010; Stulp et al., 2012, for reviews). In the domain of demography, ‘out-group threat’ is highest when the difference in size between in-group and out-group is small, especially if the size of that difference is shrinking (Hewstone et al., 2002; Slack & Doyon, 2001).

Demographic structure may directly influence violent competition between groups: Slack and Doyon (2001), for example, apply event history analysis to war crimes data from the former Yugoslavia (1990–1993) and find that civilian-involved acts of violence – mostly Bosnian Serbs harming Bosniak Muslims – were more likely to occur in areas with higher ‘indices of ethnic competition’ – i.e. in areas where Serbs and Muslims had similar population sizes, and in areas where the Muslim population was experiencing higher growth relative to the Serb population. Demographic changes can also lead to indirect competition, or a biased legal structure that favours in-group members, which then leads to further hostilities. White and Zoabi (2012), for instance, argue that right-wing Israelis speak transparently of ‘the demographic problem’, or ‘the fear that an Arab population in the country will become bigger than the Jewish population’ (Lustick, 2013: 185), and that demography has thus exerted a powerful influence on Israeli policies – e.g. where obtainment of nationality is facilitated only for people of specific ethno-religious backgrounds. In contrast, members of a high-status or demographically prominent group may often show magnanimity towards minority group members (Hewstone et al., 2002), when the status gap is very wide and when the minority group is small enough to not pose a threat to power relations.

In sum, we might expect parochial altruism to occur most notably in situations where: (i) behaviour is conditioned on group-level valuations, rather than individual-level characteristics; and (ii) contextual factors (e.g. norm differences, wealth/power differences, or demographic patterns) lead to negatively valenced group-level valuations.

### 1.4. A case study of inter-group relations in rural Colombia

In the present paper, we draw on a case study of two rural Colombian communities to elucidate how parochial altruism may be modulated by contextual factors, especially *perceptions* of between-group wealth differences – which may affect the extent to which out-group members are held as competitors to the in-group or as brethren in need of generosity. Both communities studied here are composed of Afrocolombian and Indigenous Emberá residents. While one community (the *inland* community) lies on the Afrocolombian–Emberá ethnic boundary, the other (the *coastal* community) is located where Afrocolombians are demographically predominant. At the district-level, Afrocolombians and Emberá at the inland site have similar group sizes, resource holdings, and power. In contrast, in the coastal district, the Emberá have a smaller group size, smaller resource holdings, and less power. These differences in the locally salient cultural context of between-group interactions allow us to explore contingency in the expression of parochial altruism.

We explore inter-group relationships in Colombia using a combination of ethnographic, social network, and economic game data. Our mixed-method approach allows us to measure expression of in-group favouritism, out-group exploitation, and out-group animus at an individual and community level. We discuss how perceptions of inequality affect whether individuals respond to out-group need with beneficence rather than contempt. Finally, we comment on the implications of our findings to the literatures linking identity-group diversity and socio-cultural outcomes via the mechanism of parochial altruism.

## 2. Inter-ethnic relationships in rural Colombia

### 2.1. Historical context of inter-ethnic relationships

The contemporary ethnic make-up of Colombia is heavily influenced by colonisation and the slave trade (Cantor, 2000; Wade, 2002; Castillo & Abril, 2009). During the late 1500s through the 1800s, Spanish colonisers transported hundreds of thousands of enslaved Africans to Colombia in order to replace the labour being performed by the (rapidly declining) enslaved Indigenous populations (Benson Latin American Collection 1779–1852; Murillo Urrutia, 2015). These enslaved individuals laboured primarily in gold and emerald mines, plantations, and cattle ranches – most notably on the Pacific coast in the states of Chocó and Cauca, which today remain areas with a strong demographic prominence of Afrocolombians (Murillo Urrutia, 2015; Wade, 2002).

After the cessation of slavery in Colombia, the relationship between the descendants of enslaved Africans and Indigenous peoples of the Pacific coast could be generally characterised as one of tolerance (Ross et al., 2015) – and sometimes even one of explicit cooperation and inter-dependence through institutions like *compadrazgo* or godparenthood (Cayón, 1973). At the national level, there remains an overarching sense of solidarity between these groups, as they have jointly fought for greater representation, visibility, and institutional support inside of Colombia (Castillo & Abril, 2009; Iglesias, 2018). However, the nature of this inter-ethnic relationship appears sensitive to local contexts, especially in recent times as competing claims over resource access and land titling have led to disagreements between some Afrocolombian and Indigenous groups (Ng'weno, 2000; Davis, 2002; García, 2009; Velasco, 2011).

Inter-ethnic relations can also be quite variable within communities: ethnographic accounts from Cayón (1973) – writing almost 50 years ago about inter-ethnic relations at the inland community described in Section 2.2 – indicate both that Afrocolombians and Emberá have long lived in a *simbiosis cultural* (with Afrocolombians, for example, commonly giving food and lodging to Emberá) and that it was also common to hear derogatory cross-group stereotypes voiced by members of both groups. He even notes cases of mistrust boiling over into inter-ethnic homicide. Ethnographic observations at the present time remain remarkably consistent with those of Cayón (1973); inter-ethnic food sharing and lodging are still daily occurrences among some – but not all – members of the community, and derogatory cross-group stereotypes are still voiced by some. These discrepant ethnographic accounts of inter-group relations raise the question as to how one can more formally assess the overall extent of inter-group cooperation and/or animus within and between communities.

### 2.2. Collaborating communities

In the coastal and inland communities considered here, a large proportion of residents – Afrocolombian and Emberá alike – are internally displaced persons (Oyola, 2015; Escobar, 2003), forced from their natal communities by guerilla and paramilitary groups, mostly in the 1990s and 2000s (Ibáñez, 2009). The coastal community is located in the Pacific region of western Colombia and relies on a mixture of artisanal fishing and local wage labour. The inland community is located in the rainforest of western Colombia and relies on a mixture of horticulture and local wage labour.

The socio-economic situation of the *in-sample* Emberá is similar in both communities: they are a demographically smaller group that resides on more marginal land than the *in-sample* Afrocolombians, and they have comparably less access to resources like electricity, clean water, and sanitation services. However, out-of-sample, at the larger administrative-district scale, the inland Emberá community is – in contrast to the coastal Emberá community – comparably well off. The inland Emberá community has a large population size, access to markets for selling hand-crafted artisanal jewelry, and key organisational connections; additionally, most individuals (though not those in sample) reside on a sovereign *resguardo*. The general status of the inland Emberá at the district level has led many inland Afrocolombians to think that the Emberá are generally well off compared with Afrocolombians, even though this is not necessarily true of the in-sample Emberá respondents, who report not having the same level of economic stability as the *resguardo* community. The perception that the Emberá are well off is virtually absent from the coastal community, however, where the Emberá population is, and is perceived to be, living under tougher socio-economic circumstances – on the border of a landfill, after a series of displacements.

**Figure 1.**
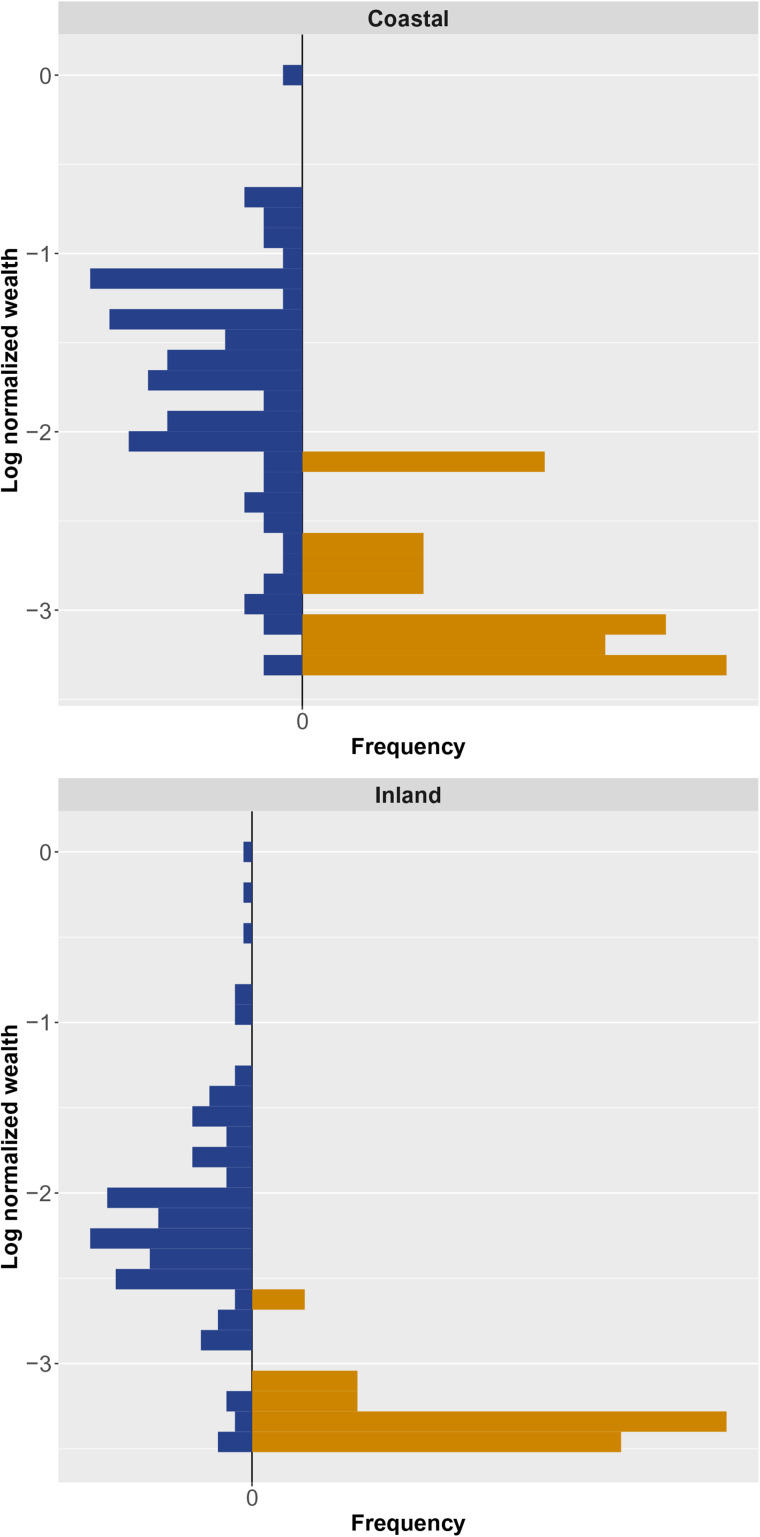
Mirror-histograms of log wealth by site and ethnic group (Afrocolombians in blue, Emberá in goldenrod). In both sites, Afrocolombians have higher average wealth than Emberá. However, even though there is less wealth overlap at the inland site, it is common for Afrocolombians there to perceive Emberá as being comparably well off, largely because they overgeneralise based on the economic status of Emberá living in the nearby *resguardo*.

At the district level, the coastal study area is predominately Afrocolombian (0.84 Afrocolombian, 0.09 Emberá and 0.07 Mestizo), while the inland study area rests along a three-way ethnic boundary with a less discrepant distribution of ethnic groups (0.14 Afrocolombian, 0.34 Emberá and 0.52 Mestizo) (DANE, 2005). The demographic composition of each sample, however, is somewhat different from that of the larger district. In both study communities, the sample is composed of one comparatively large Afrocolombian sub-community (*n* = 88 adults coastal, *n* = 130 adults inland) and one comparatively small Emberá sub-community (*n* = 28 adults coastal, *n* = 21 adults inland). In both coastal and inland communities, in-sample Afrocolombians have higher material wealth (average household-level wealth is about 3.7 times higher for Afrocolombians relative to Emberá in both communities), higher incomes (self-reported monthly income is about 1.8 and 3.9 times higher for Afrocolombians relative to Emberá in the coastal and inland communities, respectively), and stronger political influence at the local level. See Figure 1 for group-specific log-wealth distributions.

### 2.3. Research goals

To address the open theoretical and methodological questions outlined in the introduction, we ask:

Q1: *To what extent does ethnic identity structure social relationships, and behaviour in network-structured economic games measuring altruistic giving, taking/exploitation, and costly reduction.*

While both communities are characterised by ethnic diversity, the ethnographic account discussed above (Cayón, 1973) suggests that such boundaries may not actually limit cooperative giving, at least among a subset of the population. This being said, the same ethnographer notes anecdotal evidence of inter-group conflict and negative inter-group stereotypes (Cayón, 1973). To resolve these conflicting verbal accounts, we use economic games, self-reports, and ethnographic data to study the extent to which ethnic identity acts as a container for cooperation and animus. We evaluate this question both at the level of community – by contrasting network-level characterisations of inter-ethnic relations at the coastal and inland sites – and at the level of the individual – by visualising individual-level variation in preferences for in-group vs. out-group exploitation.

Q2: *How responsive is parochial altruism to varying cultural contexts?*

Specifically, if parochial altruism is indeed flexible across sites, the literature suggests that within-group cooperation and between-group animus should be more pronounced: (i) in areas, such as the geographic boundaries between groups, where norm differences, the social salience of ethnicity, and preferences for identity-group-based assortment tend to be higher (McElreath et al., 2003); (ii) in areas where the relative population size of interacting groups is more balanced (Slack & Doyon, 2001; Posner, 2004; Advani & Reich, 2015); and (iii) in areas where wealth and power differences map onto ethnic identity groups (Waring, 2012; Waring & Bell, 2013; Bell & Moya, 2021). Comparing the coastal and inland communities, we investigate if differences in the expression of parochial altruism can be attributed to these mechanisms. If such differences cannot be explained with extant models, we will consider other explanations.

Q3: *To what extent can apparent parochial altruism be explained by individual and dyadic covariates?*

Across real-world populations, inter-personal relationships are influenced by many variables – e.g. kinship and relative socio-economic status – that have the potential to covary with markers of ethnicity. For example, if individuals are more likely to give to kin than to non-kin, and kin are of the same ethnicity, then apparent in-group biases may be epiphenomenal, i.e. it may appear that giving is directed toward co-ethnics even if ethnic identity *per se* affords no special consideration in resource transfers. As such, in the Colombian context, we ask if estimates of parochial altruism are robust to controls for individual-level characteristics (e.g. wealth and food security), as well dyad-level characteristics (e.g. kinship and marriage).

## 3. Methods

**Figure 2.**
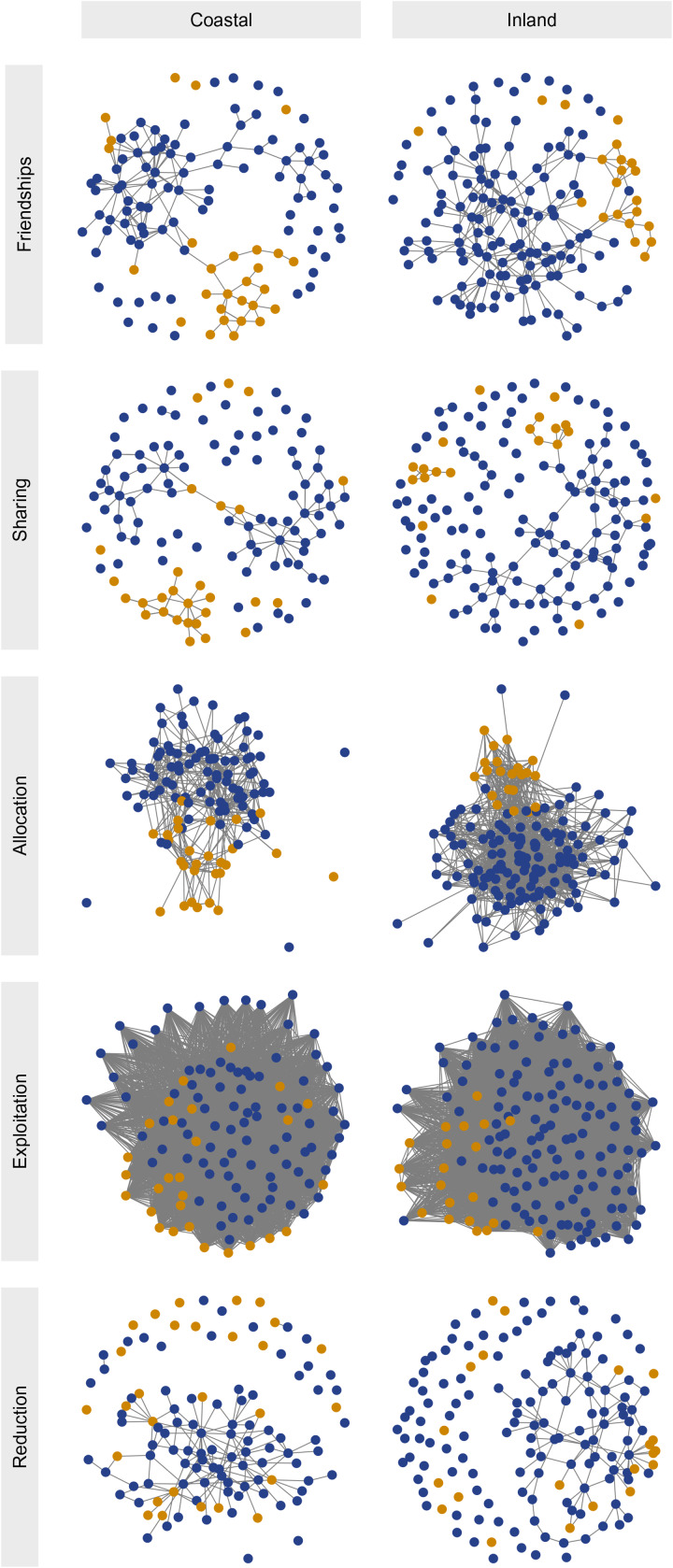
Network data. Afrocolombians are plotted in blue and Emberá in goldenrod. Quantitative estimates of assortment metrics can be found in Figure 3. The exploitation/taking game data are dense; ties represent coins left for alters, i.e. non-exploitative ties.

Dyadic data were first collected on self-reported friendships and resource transfers using ‘name-generator’ interviews (Redhead et al., 2023). Data on friendship ties were elicited by asking respondents to name the people that they had spent the most time socializing with in the 30 day period prior to the interview. Data on food/money transfers were elicited by asking respondents to name: (i) the people that they had given food/money to; and (ii) the people they had received food/money from in the 30 day period prior to the interview. Dyadic ties in giving, taking/exploitation, and costly reduction were assessed using the ‘RICH games’ of Gervais (2017), explained in Section 3.1. Network data are visualised in Figure 2.

In both sites, informed consent was obtained from each respondent prior to data collection, and from the community leader or local community council, when appropriate. Owing to limited literacy rates, informed consent was obtained verbally after providing respondents with a verbal description (in Spanish) of the research process and explaining how their data will be used (anonymously, for research purposes); in addition, participants were provided with a written consent document for their own reference. All field protocols were approved by the Department of Human Behavior, Ecology and Culture at the Max Planck Institute for Evolutionary Anthropology in Leipzig Germany. See SI, Section 2 for details on data and permissions.

### 3.1. Network-structured economic games

To measure inclinations towards parochial altruism, we used three network-structured economic games (Gervais, 2017): a giving game, a taking game and a costly reduction game. For each of these games, we presented individuals with a photo array containing 7 × 10 cm photographs of all male and female adults residing their respective communities. In total there were 115 and 151 targets/alters (in each site, respectively) to whom focal players could allocate coins or tokens. Photographs were organised onto four boards. The order of the boards was randomised between respondents, and the order of the photographs on the boards was randomised on four separate occasions over the course of data collection at each site. In total, 93 of 115 (coastal) and 137 of 151 respondents (inland) completed the economic games. All three games were played in sequence – in the same order – during the same interview. After all interviews were complete, all game participants were given the currency allocated to them by themselves and other community members during the games. The total stakes per person amounted to 83,000 (coastal) and 110,500 (inland) Colombian pesos (average of ~35 USD) at the time of data collection.

In the giving game, the stakes were set at 15,000 and 20,000 Colombian pesos in the coastal and inland sites, respectively. Individuals could allocate any number of 1,000 peso coins to any cell in the photo array, including their own. Individuals varied widely in how much was kept and how much was given, with mean giving rates of 11,760 (78.4%, coastal) and 14,870 pesos (74.3%, inland), medians of 13,000 (86.6%) and 17,000 pesos (85%), standard deviations of 3,500 and 5,000 pesos, minima of 0 and 0 pesos, and maxima of 15,000 and 20,000 pesos.

In the taking game, the stakes were set at 57,500 and 75,500 Colombian pesos. Individuals could take or leave the single 500 peso coin that was pre-allocated to each photo in the photo array. Again, individuals varied widely in how much was taken and how much was left, with mean leaving rates of 39,800 (69.2%, coastal) and 36,300 (48%, inland) pesos, medians of 47,000 (81.7%) and 34,000 (45%) pesos, standard deviations of 17,600 and 24,900 pesos, minima of 0 and 0 pesos, and maxima of 57,500 and 75,500 pesos.

In the costly reduction game, the stakes were set at 10,000 and 15,000 Colombian pesos. Individuals could keep the coins or use them purchase red tokens to punish/reduce other community members. Each token cost 1,000 pesos, and led to a reduction of the target's income by 4,000 pesos – the same multiplier used elsewhere (Gervais, 2017). Reduction was fairly infrequent, with mean payment rates for reducing of 1,600 (16%, coastal) and 1,400 (9%, inland) pesos, medians of 0 and 0 pesos, standard deviations of 2,800 and 3,400 pesos, minima of 0 and 0 pesos, and maxima of 10,000 and 15,000 pesos.

### 3.2. Statistical analysis and software

Network data collection and entry was managed using a custom R package (Ross & Redhead, 2021, 2023) and data analysis was handled entirely in R (version 4.0.5, R Core Team, 2021). Statistical models were coded in Stan and fit using the rstan package (version 2.21.2, Stan Development Team, 2020). We checked model fit and Markov Chain Monte Carlo performance using trace plots, 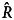, reported effective samples, and a variety of other visualisations (Gabry et al., 2019). Our statistical models, outcome and control variables, and data collection protocols are defined precisely in the SI. To model network data, we use a generalisation of the Social Relations Model (Kenny & La Voie, 1984; Koster et al., 2019; McElreath, 2016) to multinomial outcome data (see SI, Section 3).

#### 4. Results

### 4.1. Quantitative findings

**Figure 3.**
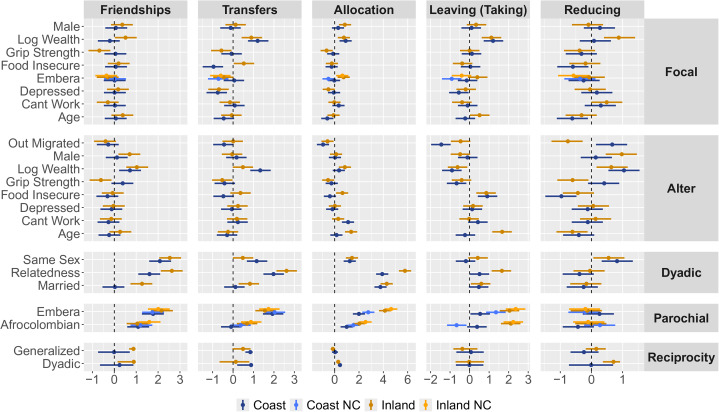
Multinomial regression results (standardised coefficients) from the Social Relations Model. Points and line-ranges show the standardised effects of predictor variables on outcomes (as medians and 90% credible intervals). When the credible intervals exclude the value of zero (plotted as a dashed vertical line), there is evidence of a reliable effect. Each column indicates an independently modeled outcome variable: (i) friendship/socialising ties, (ii) food/money transfers; (iii) coin allocations in the allocation game; (iv) coin deductions in the taking game (coded so that positive parameter estimates reflect *leaving* coins); and (v) coins paid to reduce alters in the costly reduction game. For each of these outcomes in each community, we fit two models: both included the predictors directly related to parochial altruism (e.g. as in row 4), but the first (NC; *no controls*) excluded control variables – that is, the predictors in all other rows – and the second included all controls. The key estimates of interest are shown in the ‘Parochial’ row. For example, in the allocation game, both Afrocolombian and Emberá individuals (in both sites) showed a reliably positive tendency to give more to co-ethnics. Likewise, in the taking game, Emberá individuals, as well as inland Afrocolombian individuals, showed a reliably positive tendency to leave more for co-ethnics. However, coastal Afrocolombian individuals showed a reliable tendency to leave more for the ethnic out-group (model with no controls; light blue) or no tendency for preferential out-group exploitation (model with controls; dark blue).

The results of model fitting are visualised in Figure 3. Within each column, blocks show the standardised effects of focal, alter, and dyadic characteristics on the likeliness of a tie. Of principal interest to our research questions are the effects in the *Parochial* block (row 4). Parameter estimates are blue for the coastal community and goldenrod for the inland community. Light goldenrod and light blue bars plot estimates from models without control variables – i.e. models that included the ethnicity of the focal and alter, with no other covariates. Dark goldenrod and dark blue bars give the same estimates while accounting for the full set of controls – that is, all of the predictors listed in the figure. The effects of control variables are similar to those described in Pisor et al. (2020) and are largely similar between communities (see SI, Section 4 for additional details).

Q1: *To what extent does ethnic identity structure social relationships, and behaviour in network-structured economic games measuring altruistic giving, taking/exploitation, and costly reduction*

Friendship ties suggest a high degree of social assortment on the basis of ethnic group identity; this parallels similar findings in other countries (e.g. Power, 2017; Baerveldt et al., 2007). Despite Afrocolombians and Emberá living in close proximity to each other in both communities, socialising is primarily confined to within-ethnicity interactions. These data correspond to historic accounts of a paucity of inter-ethnic marriages despite a long history of social contact (Cayón, 1973), and genetic evidence that shows a high degree of population substructure in the Pacific region of Colombia, in contrast to the Caribbean region where genetic admixture is higher (Ossa et al., 2016).

From the perspective of Emberá individuals in both communities, positive ties – be they friendships, transfers of food or money, or transfers of coins in the RICH allocation and taking games – are more likely to be directed towards other Emberá than towards Afrocolombians. This social assortment is easily seen in Figure 2. Afrocolombians at the inland community also preferentially form friendships with and give to other Afrocolombians; however, this effect only partially holds in the coastal community, where food and money transfers (Figure 3, column 2), as well as behaviour in the taking game (Figure 3, column 4), show no reliable evidence of parochial preferences.

**Figure 4.**
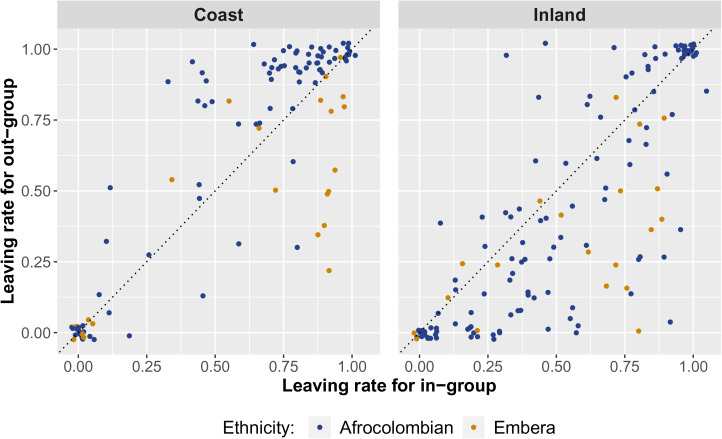
Scatterplots of the raw taking game data by site. Each point represents an individual, with the *x*-coordinate reflecting the in-group coin leaving rate and the *y*-coordinate reflecting the out-group coin leaving rate. Individuals in the upper-right corner of the plot showed indiscriminate charity, and refused to exploit others. Individuals in the lower-left indiscriminately took from all others. Individuals above the diagonal line showed a preference for leaving coins for the ethnic out-group at higher rates than the ethnic in-group, and *vice versa* for the individuals below the diagonal line. Extensive individual-level heterogeneity is apparent. At the coastal site, most Afrocolombians exhibit a preference to avoid taking from the out-group at a higher rate than the in-group. In contrast, at the inland site, the majority of individuals took from the ethnic out-group at higher rates.

Although ethnic group membership clearly structures positive social relationships and allocation game play at both sites, there is less evidence of structure in networks reflecting negatively valanced relations. There is no clear ethnic pattern of costly reduction (Figure 3, column 5) directed at either in-group or out-group members in either community. Additionally, play in the taking game varied substantially across sites (Figure 3, column 4). Figure 4 explores this finding in more detail, and illustrates that there is substantial variation in exploitation behaviour, both across sites and ethnic identity groups, and between individuals of a given ethnic identity group within sites.

Q2: *How responsive is parochial altruism to varying cultural contexts?*

When comparing measured parochial altruism at the coastal and inland communities (Figure 3, row 4), the effects in the taking game (Figure 3, column 4) and self-reported food/money sharing networks (Figure 3, column 2) stand out. In the taking game, where coins *taken* benefit the focal at the expense of an alter, both Afrocolombians and Emberá at the inland community are more likely to take coins from out-group members than in-group-members; however, on the coast, model estimates suggest that Afrocolombians are either just as likely to leave coins for Emberá as for Afrocolombians (model with controls) or are *more* likely to leave coins for Emberá than for Afrocolombians (model without controls). This effect can also be noted in Figure 4, as the majority of coastal Afrocolombian individuals cluster in the upper triangle of the plot, where the rate of leaving coins for out-group members exceeds the rate of leaving coins for in-group members. Similarly, Afrocolombians in the inland community appear to show parochialism in the food/money sharing network – a fact that may reflect a common (although not universal, Cayón, 1973) rejection of inter-ethnic food-sharing requests – while Afrocolombians in the coastal community show no such parochial preference and commonly engage in inter-ethnic food transfers. We discuss further qualitative evidence concerning these key findings, and provide more details about the relevant differences in cultural context, in Section 4.2.

Q3: *To what extent can apparent parochial altruism be explained by individual and dyadic covariates?*

We find that estimates of parochialism (Figure 3, row 4) are surprisingly robust to the inclusion of controls for material wealth, food security, marriage ties, and genetic relatedness – covariates that could otherwise generate epiphenomenal parochialism, especially in contexts like the RICH allocation game, where the set of resources that can be distributed is much smaller than the set of possible recipients. In one case to the contrary, the taking game model without controls appears to suggest a case of ‘anti-parochialism’, where Afrocolombians in the coastal community preferentially leave coins for Emberá alters. This effect, however, is attenuated in the model that includes control variables. As these control variables include the material wealth and food insecurity of the alter, the reduction in the effect size of anti-parochialism might indicate a mediating role of economic need at the coastal site in driving transfers from Afrocolombians toward Emberá.

### 4.2. Qualitative accounts

When asked in post-game interviews to explain their rationales for taking from whom they did, it was common for coastal Emberá respondents to emphasise taking from ‘those who already have money to live on’ or ‘those who have jobs’ and leaving for ‘people in similar or worse situations to [themselves]’ and ‘[their] neighbours who are also poor’. Some coastal Afrocolombians also specifically mentioned out-group ethnicity as a motivation for *not* taking coins: ‘[I left coins for] the Indigenous, the sick, and people of old age’, grouping Emberá residents into the class of people deserving special consideration. Afrocolombians and Emberá at the coastal site both agree on whose relative need is greater; accordingly, Afrocolombians did not show evidence of parochialism in real-world food/money transfers or in experimental exploitation decisions in the RICH taking game. Qualitative responses at the coastal community focused on objective need and carried little emotional valence.

Similarly, in the inland community, Emberá participants agreed that Emberá alters were more in need than Afrocolombians and biased giving towards other Emberá accordingly. In stark contrast to the coastal community, however, it was very common in post-game interviews for inland Emberá respondents to describe taking from alters (normally Afrocolombians) specifically because those alters had not cooperated in the past, and there was clearly more social friction and negative emotional valence than in the coastal community. Inland Emberá respondents would state that they took coins ‘because those people don't cooperate with you when you ask for help’ or because ‘you are hungry and ask for a favour and they do nothing’. Though not recorded explicitly in post-game interviews, informal conversations with Afrocolombians in the inland community suggested that inland Afrocolombians frequently viewed Emberá alters from their community as being as well off as the Emberá living in the nearby *resguardo*, causing them to engage in fewer inter-ethnic need-based transfers, as is clear in both the taking game and real-world food/money transfer data.

## 5. Discussion

The findings presented here, both quantitative and qualitative, contrast in some ways with the *simbiosis cultural* reported by Cayón (1973). By integrating community-wide self-report and economic game data, along with qualitative debriefing interviews and standard ethnography, we have been able to build a more representative understanding of inter-group relationships in these communities. On the one hand, the characterisation of inter-group relationships as a *simbiosis cultural* remains valid, as direct hostilities between groups are quite rare, costly reduction was not influenced by group identity, and inter-group cooperation occurs between specific individuals on an almost daily basis. However, on the other hand, social relationship and experimental transfers do exhibit signs of within-group favouritism. Lastly, the context of inter-group interactions seems to matter, as there is evidence of preferential out-group exploitation in only one of two sites.

### 5.1. The cultural context of interactions: resource competition, norm differences, and demographics

In the coastal community, Afrocolombians have a larger population size, more stable land tenure, stronger local political institutions, and greater control of the means of production (i.e. fishing boats, refrigeration) than Emberá. In contrast, in the inland community on the ethnic boundary, Afrocolombians and Emberá both have substantial population sizes, stable land tenure, and more comparable bargaining power.

Although between-group resource competition is thought to be an important driver of parochialism (Bell & Moya, 2021), direct between-group resource competition is not a central feature of the cultural context at either of these two sites. This being said, resource competition *has* been cited for the breakdown of inter-ethnic cooperation between these same two ethnic groups in *other* regions of Colombia (e.g. Ng'weno, 2000; Davis, 2002; García, 2009; Velasco, 2011), and there is much greater scope for such competition at the inland site where population size and institutional power are more balanced (Slack & Doyon, 2001; Posner, 2004).

Likewise, the contrasting inter-group relationships at the coastal and inland sites is concordant with the predictions of some models of inter-ethnic coordination games (e.g. McElreath et al., 2003; Advani & Reich, 2015). These models suggest that when each group has a large enough population size, within-group interactions will occur frequently enough to maintain distinct sub-populations with their own behavioural norms (Bunce & McElreath, 2017, 2018). If there are greater benefits to interacting with others who share the same norms (McElreath et al., 2003), then social and resources transfer networks should appear more ethnically differentiated at the inland site. However, there is no direct ethnographic indication that norm coordination is more difficult at the inland site; in fact, across study sites and ethnic groups, there is a single widely shared norm that resource transfers should be based on relative need. What appears to differ across sites is simply who is *perceived* to have more need.

### 5.2. Perceived inequality, perceived need and inter-group relations

In both communities, formal statistical analysis and qualitative post-experiment interviews identified a key norm governing transfers: *take from those who are better off and can afford it, and leave for those who are worse off and need the money more*. This is a classic need-based heuristic found across a variety of cultural groups (e.g. Peterson, 1993; Hooper et al., 2015; Aktipis et al., 2016; Hao et al., 2015; Gervais, 2017; Cronk et al., 2019). This need-based norm appears more salient to Colombian respondents than a norm for simply favouring in-group members.

We do note, however, that our full results are not consistent with any single norm driving transfers. First, although log-wealth and food security affected behaviour in the taking and costly reduction games in a way that was consistent with qualitative accounts (i.e. wealthier individuals were more likely to be taken from and reduced, while food insecure individuals were less likely to be taken from or reduced), behaviour in the positively valanced networks was somewhat different, with wealthier individuals being more likely to be nominated as friends and targets of resource transfers. Although need-based sharing *is* common, reciprocation among people with higher wealth is also common, especially in the coastal fishing community, where sharing helps to buffer stochastic fluctuations in fishing returns. In such contexts, establishment of social connections with wealthy and high-status individuals can be beneficial to a focal individual, as such connections are better able to buffer risk and improve one's socio-economic standing over long time-scales (e.g. von Rueden et al., 2019).

Although the emic perspective on need-based transfers may, at first, appear to conflict with evolutionary explanations for cooperative behaviour, need-based transfers are consistent with both individual-level models of tolerated theft (Winterhalder, 1997) and group-level models of risk-pooling (Hao et al., 2015). Imbalance in the marginal fitness benefits of some unit of food, money, or other resource has the potential to lead to conflict, as a resource-poor individual may be willing to escalate their demands for a unit of resource from a resource-rich individual; at an individual-level, this dynamic can lead to need-based transfers, whereby a well-off giver shares a resource whose benefit to an impoverished receiver exceeds the cost to the giver of defending that resource (Jones, 1984; Winterhalder, 1996, 1997; Rusch, 2014). At a larger scale, cultural institutions based around need-based transfers may lead to more optimal risk pooling and more resilient groups (Hao et al., 2015).

In contexts of ethnically structured inequality in wealth or power, individuals in underprivileged groups may be especially likely to direct aid to coethnics (Waring, 2012; Waring & Bell, 2013). Although not true across all ethnic boundaries, if ethnicity and need are perceived to covary, then members of a relatively well-off ethnic group may use ethnicity as a heuristic to direct need-based transfers, attenuating the overt expression of parochialism (see also Rusch, 2014). In the coastal community, Emberá are a small proportion of the population and there is large between-group, but little within-group variation in wealth; ethnicity thus covaries strongly with perceived need. Coastal Afrocolombians and Emberá both recognise that the social obligation to help the needy means that resources should flow towards Emberá. In the inland community, however, district-level population sizes are more balanced, and within-group variation in wealth and status (e.g. comparing in-sample Emberá with those living on the *resguardo*) is higher. Here, ethnicity does not covary with perceived need and thus fails serve as an indicator that can be used to guide transfers. As such, both inland Afrocolombians and Emberá see themselves as needier, and are likely to cite need when directing resources towards members of their own groups. Our findings here echo Hewstone et al. (2002): members of a high-status group may be likely to show magnanimity when the status gap is very wide (i.e. when it is clear to all members of both groups who is most in-need).

### 5.3. Rethinking the theoretical linkages between diversity and socio-cultural outcomes

Perhaps influenced by a rash of inter-ethnic and sectarian conflicts (McGarry & O'leary, 2013), some social scientists in the 1990s and 2000s came to speculate that identity-group diversity might drive many adverse socio-cultural outcomes (see Patsiurko et al., 2012, for an overview). Such arguments relied – either implicitly or explicitly – on assumptions of parochial altruism, i.e. that individuals in a particular group will cooperate amongst themselves, while competing with other groups, in turn causing more internal discord in territories where identity-group diversity is high.

Reviewing the economics and political science literature, Patsiurko et al. (2012), found that the effects of cultural homogeneity vs. diversity on socio-cultural outcomes were explored in studies of national economic success (Easterly & Levine, 1997), provisioning of public goods (Alesina et al., 1999; Habyarimana et al., 2009), nationalist insurgencies (Cederman & Girardin, 2007), civil wars (Fearon et al., 2007; Wimmer et al., 2009; Cederman et al., 2010), and war crimes (Slack & Doyon, 2001), among other topics. In these studies, diversity is indeed frequently found to be *correlated* with adverse socio-cultural outcomes (e.g. higher conflict rates, lower public goods provisioning, or more frequent war crimes) in aggregate-level data, but such findings are not universal (e.g. see Ottaviano & Peri, 2006).

In a similar review of the organisational studies literature, Waring and Bell (2013) found that ethnic diversity was frequently associated with reduced cooperation at both a community (e.g. Banerjee et al., 2005; Miguel & Gugerty, 2005; Ruttan, 2006; Bardhan et al., 2007) and interpersonal level (e.g. Williams & O'Reilly III, 1998; Pitts & Jarry, 2007; Hur, 2013; Pelled et al., 1999; Watson et al., 1993). Waring and Bell (2013) note, however, that this literature is also not monolithic, and that evidence is often mixed or varied, with diversity sometimes having positive effects on socio-cultural outcomes (e.g. informational diversity can improve group performance in complex tasks; Jehn et al., 1999).

Increasingly, researchers (e.g. Waring & Bell, 2013; Wimmer et al., 2009) are beginning to argue that one must be careful to distinguish correlations between diversity and socio-cultural outcomes, from causal effects of diversity on such outcomes – especially in cases where studies are based on non-experimental, ecological regression designs. Analyses of aggregate-level variables are often subject to confounding (Wakefield, 2004; Waring & Bell, 2013) and spurious associations have been used as rhetorical justification for everything from arcane legal policies to direct violence (Patsiurko et al., 2012; McGarry & O'leary, 2013). Our finer-scale findings, much like those of Waring and Bell (2013), serve to question the idea that parochialism is ubiquitous; instead it appears to be context dependent and linked to perceptions of between-group differences in socio-economic status.

In addition to the potential validity concerns inherent in ecological regression designs, there are also potential issues related to sample selection bias. Fearon and Laitin (1996: 716), for example, note that much of the research on intergroup relations focuses on cases of between-group conflict, essentially selecting on the dependent variable and making such conflict seem ubiquitous: ‘violence is assumed to follow ethnic tensions as night follows day’. Moreover, ‘the salience and extremity of intergroup hostility’ to researchers can lead to a literature in which ‘the study of intergroup relations is equivalent to the study of intergroup conflict’ (Brewer, 2010: 535). Our methods, if deployed broadly across a representative sample of cultural groups (e.g. study sites not selected on the basis of inter-group conflict), would permit a more accurate estimate of the prevalence of parochial altruism. While there are many between-group boundaries at which conflict could take place, the fraction of boundaries where conflict actually *does* take place appears to be much smaller (Fearon & Laitin, 1996).

In light of such issues, we argue that more precision is needed when discussing the mechanisms through which identity-group diversity may be linked to adverse socio-cultural outcomes. Alesina and Ferrara (2005: 762), for example, frame their work as exploring the costs and benefits of *diversity* on socio-cultural outcomes, stating:

The potential costs of *diversity* are fairly evident. Conflict of preferences, racism, and prejudices often lead to policies that are at the same time odious and counterproductive for society as a whole. The oppression of minorities may lead to political unrest or even civil wars. But a diverse ethnic mix also brings about variety in abilities, experiences, and cultures that may be productive and may lead to innovation and creativity.

However, it is notable that while conflicting preferences, racism, prejudice, and oppression of minorities might all serve as causes of adverse socio-cultural outcomes, none of these variables are essential characteristics of a diverse society. Thus, it is important that when researchers consider the policy implications of their work, they focus attention on the relevant variables – that is, if researchers believe racial biases or unfair social structures that oppress minorities to be fundamental causes of political unrest, then they should focus precisely on those variables, rather that referencing ‘diversity’ more broadly.

The fact that there might be more scope for prejudice in more diverse locations does not imply that diversity *per se* has costs, and – in fact – if diversity carries positively externalities (as Alesina and Ferrara, 2005 suggest), then diverse societies may be especially successful when formal and informal structures of racism, prejudice and oppression are attenuated – e.g., see Jha (2013) on the mutual benefits conferred by inter-group trade. Beheim and Bell (2024) formalise this idea, and show that diversity can increase group-beneficial outcomes over a range of interaction payoff structures, especially interactions featuring complementarities between actors.

#### 5.3.1. The value of social networks and network-structured economic games for studying human behaviour cross-culturally

By deploying robust, mixed-methods research protocols that integrate social network data, an informative set of dyadic economic games, and qualitative post-game interviews, we were able to measure in-group favouritism separately from out-group exploitation or animus (Pisor & Ross, 2023). This methodologically pluralistic approach has allowed us to cross-check our inferences and show that our findings are not reducible to artefacts of a single method of data collection. Likewise, our methods allow us to control for a suite of important focal, alter, and dyadic covariates that might confound estimates of the effects of ethnicity on in-group favouritism; we show that our statistical findings are robust to such controls. Within sites, triangulation across methods and consideration of confounding factors helps to establish internal validity (Pisor & Ross, 2022; Pisor et al., 2020).

Additionally, our methods facilitate standardised comparisons between sites, opening the door to tests of generalisability (Tiokhin et al., 2019) and assessments of the site-level drivers of parochial altruism. By replicating our study design across two ethnic groups at two sites, we are able to comment on the (lack of) generalisability in expression of parochial altruism (especially the out-group animus component) within the Pacific region of rural Colombia. The paucity of out-group exploitation by coastal Afrocolombians appears to be driven by perceptions of need and by norms for basing transfers on need. However, wider-scale, standardised, cross-cultural assessments are still needed to assess the extent of variation in parochial altruism, as well its causes and consequences.

In this study, we have been limited to studying average, site-level, differences in expression of parochial altruism. However, Figure 4 suggests that there are also substantial individual-level differences in expression of parochial altruism *within sites*. For example, even at the inland site – where parochialism was higher on average – we found that a substantial fraction of the Afrocolombian population left coins for the ethnic out-group at higher rates than the ethnic in-group. We hypothesise that this variation will be related to individual heterogeneity in residential patterns, rates of between-group social network connections, and ultimately dyad-level perceptions of economic need. That is, we expect Afrocolombians with social network ties to Emberá individuals, and even Afrocolombians living near Emberá households, to have better awareness of the true economic need of in-sample Emberá families, and thus be less parochial. In future work, we aim to measure perceptions of socio-economic status at the dyad level in order to test this idea.

### 5.4. Conclusions

In this paper, we sought to explore the extent to which parochial altruism may vary as a function of local socio-ecological context. Our findings are consistent with a growing body of literature suggesting that parochial altruism is not ubiquitous (e.g. see Purzycki & Lang, 2019; Hruschka & Henrich, 2013; Yamagishi & Mifune, 2016; Brewer & Caporael, 2006; Schaub, 2017; Cashdan, 2001; de Dreu et al., 2014; Böhm et al., 2020; Balliet et al., 2014; Abbink et al., 2012; Pisor & Ross, 2023), and that the context of between-group interaction can strongly impact whether cooperation breaks down in more diverse communities (Waring & Bell, 2013; Alesina et al., 2016) or whether norms and institutions emerge in order to reap the potential benefits of between-group connections (Jha, 2013; Bunce, 2021; Glowacki, 2022). While there is certainly scope for conflict in diverse societies, such adverse outcomes can probably be attenuated by minimising the structural biases that trigger perceptions of unequal status or unfair treatment.

## Supporting information

Ross and Pisor supplementary material Ross and Pisor supplementary material

## Data Availability

Code and data for diagnostics and analysis replication are provided in the Supplementary Materials and will be maintained on GitHub at: www.github.com/ctross/parochialism_and_inequality

## Supplementary material

The supplementary material for this article can be found at https://doi.org/10.1017/ehs.2024.43.
