# Supplementary material for: Perceived inequality and variability in the expression of parochial altruism
Source: Evol Hum Sci. 2025 Jan 20;7:e3. doi: 10.1017/ehs.2024.43 (PMC11810521; doi:10.1017/ehs.2024.43)
Supplement: Ross and Pisor supplementary material [file S2513843X24000434sup001.pdf]

Supplementary Materials to: “Perceived inequality and variability in the expression of parochial altruism”

Cody T. Ross[1] and Anne C. Pisor[1,2,3]

[1] Max Planck Institute for Evolutionary Anthropology. Department of Human Behavior, Ecology, and Culture. Germany.
[2] Department of Anthropology and Social Science Research Institute. The Pennsylvania State University. USA.
[3]Department of Anthropology, Washington State University. USA.

Contents

1 Notes on terminology **1**
1.1 Parochial altruism 1
1.2 Identity-groups 2
1.3 Altruism versus individual incentives . . . 2

2 Data collection **2**
2.1 Outcome data 2
2.2 Predictor variables 4
2.3 Covariates 4

3 Modeling **5**
3.1 Priors 5
3.2 Software and fitting 6

4 Model results **6**
4.1 Friendship 6
4.2 Food/money transfers 6
4.3 Allocation game 6
4.4 Leaving game 6
4.5 Reducing game 6

1. Notes on terminology

1.1. Parochial altruism

When Bowles (2008) and colleagues introduced the term ‘parochial altruism’ to the literature, they used standard definitions, stating simply that: “altruism is conferring benefits on others at a cost to oneself” and that “parochialism is favouring ethnic, racial or other insiders over outsiders”. Over the last few decades, however, as the literature has developed, there has been some debate about terminology related to ‘parochial altruism’.

First, there is debate over whether ‘parochial altruism’ is truly ‘altruistic’. Because purported ‘parochial altruists’ may sometimes personally benefit from their actions, it is not always clear if the term ‘altruism’ is warranted (West et al. 2011). Second, there has been some worry that altruism and spite might be being confounded (Krupp 2013). That is, there has been worry that harmful—technically “spiteful” (Krupp 2013)—actions towards out-group members have been labeled as ‘altruistic’ behaviors, simply because they indirectly benefit in-group members by virtue of affecting the relative fitness of in-group and out-group members. Calling a ‘spiteful’ behavior towards out-group members ‘altruistic’ might thus mischaracterize the adaptation.

On the later point, we concur with Krupp (2013) and follow Bowles (2008) in his initial use of the term ‘parochial altruism’. That is, we do not label harmful or spiteful behaviors towards out-groups as altruistic. Bowles (2008) and colleagues did not intend to classify spiteful actions towards out-group members as ‘altruistic’, they simply predicted a behavioral complex featuring both in-group altruism and out-group spite, since mathematical modeling suggested that an increase in the frequency of either of these these two variables makes the other more likely to increase in frequency as well. Like Bowles (2008), we view ‘parochial altruism’ as a *behavioral complex*, which includes altruistic actions toward in-group members and spiteful actions towards out-group members.

On the first point, definitions of parochial altruism are somewhat variable in the literature (Pisor & Ross 2023). Some authors refer to in-group *cooperation*—or the generation of benefits for in-group members at a cost to the self (e.g., Bowles 2008)—while others refer only to in-group *favoritism* (e.g., Rusch 2014), which may include situations in which in-group benefits are generated without personal costs (Bernhard et al. 2006). West et al. (2011) object to the more expansive terminology, arguing that it muddies the waters, and we agree with their reasoning. However, for completeness, we review studies of *in-group favoritism* paired with *out-group hostility* in the main text, since it is often impossible to establish whether a purportedly altruistic in-group behavior meets the stringent definition of West et al. (2011). We note also that Beheim & Bell (2024) have recently re-derived classic models linking group-beneficial behaviors and group-structure as special cases of a more general model, which represents different kinds of synergies (e.g., cooperation, coordination, anti-coordination, or complementarity) in a continuous space; they show that evolutionary dynamics are similar, even when a group-beneficial behavior is not purely altruistic. As such, while we acknowledge these debates around terminology, we continue to use the phrase ‘parochial altruism’ in our manuscript, as it is a common term in the literature.

1.2. Identity-groups

Much of the literature uses terms referencing ethnicity specifically, e.g., “ethnic diversity” or “ethnic conflict”. We instead use the term “identity-group”, as ethnicity is only a single kind of group marker, and other kinds of identity groups can structure cooperation and animus in similar ways.

1.3. Altruism versus individual incentives

Although there may be altruistic motives for participating in conflict, this is not to say that participation in conflict is always selfless, as there can be both individual-level (Glowacki & Wrangham 2013) and group-level (Zefferman & Mathew 2015) benefits for participation. Ultimately, whether participation in a conflict is altruistic in a strict sense might be unknowable in advance, frequency dependent, dependent on the extent of power asymmetries within and between groups, and variable across individuals.

2. Data collection

Data were collected at two communities in rural Colombia, which we refer to as the coastal and inland sites (see Figure 1). The data presented and analyzed here were collected as part of a wider, longitudinal field study on aspects of wealth, demography, and social networks. Informed consent was obtained from each respondent prior to data collection, and from the community leader or local community council, when appropriate. Because of sometimes limited literacy, informed consent was obtained verbally after providing participants with a verbal description (in Spanish) of the research process and explaining how their data will be used (anonymously, for research purposes); in addition, participants were provided with a written consent document. All field protocols were approved by the Department of Human Behavior, Ecology, and Culture at the Max Planck Institute for Evolutionary Anthropology in Leipzig, Germany.

Each community was sampled as completely as possible within a pre-demarcated geographic area; nearly all households in the census area opted into the study. No selection criteria were applied in recruiting subjects other than ability to provide informed consent, age, and residence within the census area. Thus, sample size was determined by the number of adult individuals residing in each community. Our full database contains several-hundred individual- and dyad-level measures collected over 11 years of fieldwork. We limited the variables included in this analysis to those described in Sections 2.1-2.3. Although we limit our presentation of results to a “base” model with no covariates, and a “full” model with all included covariates, we also conducted some exploratory analyses using only a subset of the included predictor variables. The exploratory analyses were unremarkable, and so we have limited our presentation of results to the “base” and “full” models. The full data-set is available for replication, and reanalysis using different subsets of predictors. No competent adult individuals were excluded from the sample either prior to data collection or for the purposes of data analysis; however, there were a small number of individuals who opted out of the study, and a small number of individuals who could not provide informed consent to participate in a research study (e.g., due to mental health issues or dementia) and were thus not invited to participate. We conducted all three RICH games, and report the results from all three games.

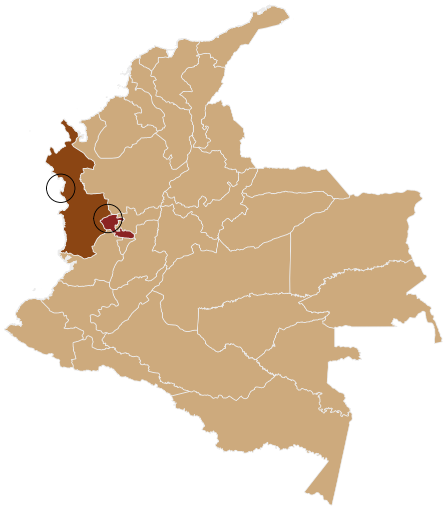

Fig. 1: Approximate location of participating communities in Colombia.

Subsistence modes and labor practices are quite variable within and across communities. The coastal community relies primarily on a mixture of fishing and local wage labor, along with hunting, horticulture, and animal husbandry. The inland community is located in the rainforests of western Colombia, and relies primarily on a mixture of horticulture and local wage labor, but hunting, fishing, and animal husbandry are also practiced, as is small-scale gold panning. Both communities are characterized by a high level of poverty relative to more urbanized areas in Colombia.

2.1. Outcome data

We measure parochial or assortative behavior using two free-recall-based social network questions:

- *Friendship ties*
  Friendship/socializing ties between each pair of individuals were assessed by asking each individual to name all individuals with whom they have spent time socializing in the last 30 days. This question was asked as part of the social network battery conducted in the winter of 2016 at the coastal site

and in the spring of 2017 at the inland site.

- *Food or money transfers*
Transfer ties between each pair of individuals were assessed by asking each individual to name all individuals to whom they have given food or money in the last 30 days, and all individuals who have given them food or money over the same time period. This question was also asked as part of the social network battery conducted in the winter of 2016 at the coastal site and in the spring of 2017 at the inland site.

We measure parochial preferences using three network-structured economic games, following the methods of Gervais (2017). Data were collected using a custom R package (Ross & Redhead 2021) to run these roster-based games. In private, each participant was presented with a game board, displaying facial photographs of all adult community members. Participants were then told that they could give coins to (in the allocation game), take coins from (in the taking/exploitation game), or pay coins to reduce the payouts of (in the reduction game) members of their community.

For each of the games, the game board consisted of a photo array containing 7x10 cm photographs of all interviewed male and female adults residing in the field-site during the winter of 2016 (or spring 2017 in the inland site). In total there were 115 alters (recipients) to whom focal players (deciders) could allocate coins or tokens (151 in the inland site). These photos were organized onto four large boards. The order of the boards was randomized between respondents, and the order of the photographs on the boards was randomized on four separate occasions over the course of data collection. In total, 93 respondents completed the economic games at the coastal site (137 at the inland site). All three games were played in sequence—in the same order (allocation, taking, and costly punishment)—during the same interview.

After all interviews were complete, all game participants were given the money that they earned during the games. Individuals who appeared as alters but who could not be found to participate as focal players (normally due to out-migration from the community) were not allocated payouts—instead, transfers directed to these players were refunded to the focal players who made such transfers. Total stakes per person at the coastal site amounted to 83,000 Colombian pesos (~27 USD at the time of data collection). At the inland site the total stake were somewhat higher: 110,500 Colombian pesos (~34 USD), due to the larger sample size in the taking game.

Expected mean payouts were set by using self-reported income at the household-level over the month prior to the initial 2016 survey in the coastal site. Assuming that 21 (five of every seven) of these days were work days, mean daily household income there is 82,700 Colombian pesos. However, there is significant inequality in income, as the median reported daily household income is only 50,900 Colombian pesos.

Game-specific details are provided below:

- *RICH allocation game*
In the allocation game at the coastal site, the stakes were set at fifteen 1,000 peso coins (15,000 pesos total). Individuals could allocate any number of these coins to any cell in the photo array, including their own. Individuals varied widely in how much was kept and how much was given, with a mean giving rate of 11,760 (78.4%), a median of 13,000 (86.6%), a standard deviation of 3,500, a minimum of 0, and a maximum of 15,000 pesos.

  In the allocation game at the inland site, the stakes were set at twenty 1,000 peso coins (20,000 pesos total). Individuals could allocate any number of these coins to any cell in the photo array, including their own. Individuals varied widely in how much was kept and how much was given, with a mean giving rate of 14,870 (74.3%), a median of 17,000 (85.0%), a standard deviation of 5,000, a minimum of 0, and a maximum of 20,000 pesos.

- *RICH taking/exploitation game*
In the taking/exploitation game at the coastal site, an initial allocation of one 500 peso coin to each photo was provided by the researcher for a total stakes of 57,500 pesos; participants could leave the 500 peso coin placed by the researcher on each photo or take it for themselves. Again, individuals varied widely in how much was taken and how much was left, with a mean leaving rate of 39,800 (69.2%) pesos, a median of 47,000 (81.7%), a standard deviation of 17,600, a minimum of 0, and a maximum of 57,500 pesos.

  In the taking/exploitation game at the inland site, an initial allocation of one 500 peso coin to each photo was provided by the researcher for a total stakes of 75,500 pesos; participants could leave the 500 peso coin placed by the researcher on each photo or take it for themselves. Again, individuals varied widely in how much was taken and how much was left, with a mean leaving rate of 36,300 (48%) pesos, a median of 34,000 (45%), a standard deviation of 24,900, a minimum of 0, and a maximum of 75,500 pesos.

- *RICH costly reduction game*
In the costly reduction game at the coastal site, the stakes were set at 10,000 pesos (ten 1,000 peso coins), which were allocated to the recipient. Individuals could keep the coins or use them purchase red tokens to punish/reduce other community members. Each token cost 1,000 pesos, and led to a reduction of the alter's income by 4,000 pesos—the same multiplier used elsewhere (Gervais 2017). Punishment was fairly infrequent, with a mean payment rate for punishing of 1,600 (16%), a median of 0, a standard de-

viation of 2,800, a minimum of 0, and a maximum of 10,000 pesos.

In the costly reduction game at the inland site, the stakes were set at 15,000 pesos (fifteen 1,000 peso coins), which were allocated to the recipient. Individuals could keep the coins or use them purchase red tokens to punish/reduce other community members. Each token cost 1,000 pesos, and led to a reduction of the alter's income by 4,000 pesos. Punishment was fairly infrequent, with a mean payment rate for punishing of 1,400 (9%), a median of 0, a standard deviation of 3,400, a minimum of 0, and a maximum of 15,000 pesos.

2.2. Predictor variables

We consider one key predictor variable for parochialism: ethnic group identity. For more precision in our inferences, we estimate parameters on the "same ethnicity" variable that are unique to ethnic group—i.e., we estimate unique effects of "same ethnicity" for Emberá and Afrocolombians.

- *Same ethnicity*
  A binary indicator if individuals i and j (that is, the decider and the recipient) are either both Emberá or both non-Emberá. If both respondents were of the same ethnicity, this value is 1; if one respondent was non-Emberá and the other Emberá, this value is 0.

2.3. Covariates

We consider eleven covariates that might play a role in explaining variation in economic game play and network ties in our statistical models. In order to normalize the effects of our shrinkage priors, we divide each of these variables by their respective maximums before model fitting. Because missing data were quite sparse, they were handled using the "mean imputation" technique: missing data were imputed a single time prior to model fitting using the mean (for continuous variables) or median (for discrete variables) of the distribution for the relevant variable.

- *Age*
  Age is typically based on self-reported date of birth. In the majority of cases, individuals know their date of birth from their national ID. In a small set of cases, especially among the elderly and Emberá sub-samples, age is only a self-reported estimate.

- *Sex*
  A binary indicator for identity as male.

- *Cant work*
  A binary indicator for if an individual is unable to work due to injury or age. This is a qualitative rating by CTR based on information/observations gleaned from interviews with each person.

- *Depressed*
  Mental health was measured using a self-report protocol based on the Kessler-6 (Kessler et al. 2002) screening scale: individuals answered a set of six questions about feelings of depression, nervousness, anxiety, tiredness, worthlessness, and hopelessness. Respondents were asked to describe how often they experienced each of these feelings during the past 30 days on a 5-point Likert-scale: "all of the time", "most of the time", "some of the time", "a little of the time", and "none of the time". We use the depression question here.

- *Food insecurity*
  Food insecurity was assessed with the question: how many days in the last month did you have so little food that you or someone in your family had go to bed hungry? Respondents indicating that someone in their household went to bed hungry for one or more days were coded as food insecure.

- *Grip strength*
  Grip strength was assessed using a Camry Digital Hand Dynamometer. Two readings were taken on each hand, and the average of all four ratings was used as our measure of grip strength.

- *Material wealth*
  As our primary measure of economic stability, we use data on the log household wealth of each focal individual in the winter of 2016 (coast) or spring of 2017 (inland). This variable is composed of (the log of) the sum total of the local monetary value of all: cars, trucks, motorcycles, mototaxis, motorboats, canoes, computers, TVs, washing machines, refrigerator, stoves, microwaves, cell phones, cows, pigs, and chickens in the household of the focal respondent.

- *Out-migration*
  A binary indicator for individuals who were present in the community in winter/spring 2016/2017, but who were not present in the community in winter/spring 2017/2018 during the economic games (and could not be found to play the games, despite appearing as alters).

- *Same sex*
  A binary indicator if individuals i and j are either both male or both female. If both respondents were of the same sex, this value is 1; if one respondent was male and the other female, this value is 0.

- *Marriage*
  Marriage ties between each pair of individuals were assessed by asking each individual to name all individuals with whom they are currently married.

- *Relatedness*

Relatedness ties between each pair of individuals were created by first asking each individual in the community to name all parents and children. A community-wide pedigree was then constructed and used to create a pairwise matrix of relatedness values.

3. Modeling

Let $A_{[i,1:J]} \in \mathbb{N}^J$ be a vector of coin allocations or network ties by individual i across J alters. We can model these outcomes using a multinomial regression model:

$$A_{[i,1:J]} \sim \text{Multinomial}(\text{Softmax}(\theta_{[i,1:J]})) \tag{1}$$

where the Softmax function maps $\theta_{[i,1:J]} \in \mathbb{R}^J$ to a unit J-simplex, which gives the probability of an allocation to each alter. To parameterize the model, we first define intermediate variables. The effects of covariates linked to a focal individual are defined as:

$$\psi_{[i]} = \lambda_{[i]} + \alpha_{[0]} + \alpha_{[1]}X_{[i]} + \alpha_{[2]}Y_{[i]} + \ldots \tag{2}$$

The effects of covariates linked to alters are defined as:

$$\phi_{[i,1:J]} = \pi_{[1:J]} + \beta_{[1]}X_{[1:J]} + \beta_{[2]}Y_{[1:J]} \ldots \tag{3}$$

And, the effects of covariates linked to dyads are defined as:

$$\kappa_{[i,1:J]} = \delta_{[i,1:J]} + \gamma_{[1]}Z_{[i,1:J]} + \ldots \tag{4}$$

We can then define $\theta_{[i,1:J]}$ as:

$$\theta_{[i,1:J]} = (\psi_{[i]} + (\phi_{[i,1:J]} + \kappa_{[i,1:J]})) \circ Q_{[i,1:J]} \tag{5}$$

Here X and Y are covariate vectors, while Z is a covariate matrix. This implies that $\psi_{[i]}$ is a scalar, and that $\phi_{[i]}$ and $\kappa_{[i]}$ are J-vectors. Finally, Q is a $J \times J$ matrix with ones on the off-diagonals and zeros on the diagonal, and serves as an indicator for *focal* and *alter* cases; in other words, Q indicates which individual is focal and which individuals are alters in each row. The symbol $\circ$ denotes the Hadamard product, which leads to the ith cell in $\theta_{[i]}$ being set to zero. As such, the coefficients on the predictor variables represent the change in log-odds of an allocation to an alter, relative to an allocation to self. The parameters λ and π are both J-vectors and serve as random effects for focal and alter, respectively. The parameter matrix δ is a dyad-level random effect.

In the allocation game model, $A_{[i,1:J]}$ represents the number of coins placed by focal individual i on the photographs of alters $1, \ldots, J$, where the photograph of individual i is included in the set of J photographs (individuals can allocate to themselves by placing coins on their own photos). In the taking game model, $A_{[i,1:J]}$ represents the number of coins left by individual i on the photographs of alters $1, \ldots, J$—this is limited by the study design to be either a single coin or nothing, with the exception of the photograph of the focal individual ($A_{[i,i]}$), who will have the sum total of coins taken from alters. In the costly punishment model, $A_{[i,1:J]}$ represents the number of punishment tokens placed by focal individual i on the photographs of alters $1, \ldots, J$—with the exception that $A_{[i,i]}$ represents the number of coins kept by individual i and not allocated to punishment.

Finally, in the friendship and food/money transfer models, $A_{[i,1:J]}$ represents the directed ties between individual i and alters $1, \ldots, J$—this is limited by the study design to be a binary indicator of a tie existing. In this outcome, $A_{[i,i]}$ is set as: $J - \sum_{j \neq i} A_{[i,j]}$; i.e., the number of ties not made to alters in the community. This reflects the empirical fact that the total number of possible ties in the outcome vector is constant across individuals in the community, and keeps the data structure of the outcomes consistent across games.

3.1. Priors

Because both models are heavily parameterized relative to the number of individuals in the sample, we use regularizing priors on all top-level parameters:

$$\alpha \sim \text{Normal}(0, 1.5) \tag{6}$$

$$\beta \sim \text{Normal}(0, 1.5) \tag{7}$$

$$\gamma \sim \text{Normal}(0, 1.5) \tag{8}$$

These priors shrink effects towards zero, reducing effective parameter complexity.

We then use the complex pooling structure of the Social Relations Model outlined in *Statistical Rethinking* (McElreath 2020):

$$\begin{pmatrix} \lambda_{[i]} \\ \pi_{[i]} \end{pmatrix} \sim \text{MV Normal}\left(\begin{pmatrix} 0 \\ 0 \end{pmatrix}, \begin{pmatrix} \sigma_\lambda^2 & \sigma_\pi\sigma_\lambda\rho \\ \sigma_\lambda\sigma_\pi\rho & \sigma_\pi^2 \end{pmatrix}\right) \tag{9}$$

which, computationally (Stan Development Team 2020), is better to implement by defining:

$$\begin{pmatrix} \lambda_{[i]} \\ \pi_{[i]} \end{pmatrix} = \begin{pmatrix} \sigma_\lambda \\ \sigma_\pi \end{pmatrix} \circ \left(L * \begin{pmatrix} \hat{\lambda}_{[i]} \\ \hat{\pi}_{[i]} \end{pmatrix}\right) \tag{10}$$

$$\sigma_\lambda \sim \text{Exponential}(1.5) \tag{11}$$

$$\sigma_\pi \sim \text{Exponential}(1.5) \tag{12}$$

$$\hat{\lambda}_{[i]} \sim \text{Normal}(0, 1) \tag{13}$$

$$\hat{\pi}_{[i]} \sim \text{Normal}(0, 1) \tag{14}$$

$$L \sim \text{LKJ Cholesky}(2) \tag{15}$$

where L is a Cholesky factor from the decomposition of the 2×2 correlation matrix with ρ on the off-diagonal.

We use this same approach for the dyad-level random effects:

$$\begin{pmatrix} \delta_{[i,j]} \\ \delta_{[j,i]} \end{pmatrix} = \begin{pmatrix} \sigma_\delta \\ \sigma_\delta \end{pmatrix} \circ \left(L_\delta * \begin{pmatrix} \hat{\delta}_{[i,j]} \\ \hat{\delta}_{[j,i]} \end{pmatrix} \right) \quad (16)$$

$$\sigma_\delta \sim \text{Exponential}(1.5) \quad (17)$$

$$\hat{\delta}_{[i,j]} \sim \text{Normal}(0, 1) \quad (18)$$

$$L_\delta \sim \text{LKJ Cholesky}(2) \quad (19)$$

Under this model, ρ gives an indication of generalized reciprocity—i.e., do those who give more (to anyone) also receive more (from anyone)?—and ρ_δ gives a measure of dyadic reciprocity—i.e., if focal i gives to alter j, then is j also more likely to give to i?

3.2. Software and fitting

Data analysis was handled entirely in R (R Core Team 2021). Statistical models were coded in Stan and fit using the `rstan` package (Stan Development Team 2020). We diagnosed model fits and Markov Chain Monte Carlo performance using trace plots, $\hat{R}$, and reported effective samples. See Figures 3-6 for a sample of trace plots from each model. All diagnostics indicated good model fit. Code and data for diagnostics and analysis replication are provided in the Supplementary Materials and will be maintained on GitHub at: www.github.com/ctross/parochialism_and_inequality

4. Model results

In the main text, we plot the results from the model which included log-wealth. Here, we provide a robustness check where we include wealth on an absolute scale. Figure 2 here thus replicates the analysis presented in Figure 3 of the main text, changing only "Material Wealth" to "Log Material Wealth". Estimates are provided as Bayesian posterior-distribution medians and 90% credible intervals. Our findings are robust to using either material wealth or log material wealth as a predictor.

4.1. Friendship

Tables 1–4 provide estimates of the predictors of friendship nominations.

4.2. Food/money transfers

Tables 5–8 provide estimates of the predictors of reported food/money transfers.

4.3. Allocation game

Tables 9–12 provide estimates of the predictors of allocation game money transfers.

4.4. Leaving game

Tables 13–16 provide estimates of the predictors of taking/leaving game money transfers. Parameter estimates reflect slopes of the probability of *leaving* money for another person, although the game was framed as a *taking* game.

4.5. Reducing game

Tables 17–20 provide estimates of the predictors of costly reduction.

References

Beheim, B. A. & Bell, A. V. (2024). Why cultural distance can promote–or impede–group-beneficial outcomes. *Evolutionary Human Sciences*, *6*, e14.

Bernhard, H., Fischbacher, U. & Fehr, E. (2006). Parochial altruism in humans. *Nature*, *442*, 912–915.

Bowles, S. (2008). Conflict: Altruism's midwife. *Nature*, *456*, 326–327.

Gervais, M. M. (2017). RICH economic games for networked relationships and communities. *Field Methods*, *29*, 113–129.

Glowacki, L. & Wrangham, R. W. (2013). The role of rewards in motivating participation in simple warfare. *Human Nature*, *24*, 444–460.

Kessler, R. C., Andrews, G., Colpe, L. J. et al. (2002). Short screening scales to monitor population prevalences and trends in non-specific psychological distress. *Psychological Medicine*, *32*, 959–976.

Krupp, D. B. (2013). How to distinguish altruism from spite (and why we should bother). *Journal of Evolutionary Biology*, *26*, 2746–2749.

McElreath, R. (2020). *Statistical rethinking: A Bayesian course with examples in R and Stan*. Chapman and Hall/CRC.

Pisor, A. C. & Ross, C. T. (2023). Parochial altruism: What it is and why it varies. *Evolution and Human Behavior*, (pp. 1–15).

R Core Team (2021). *R: A Language and Environment for Statistical Computing. V. 4.0.5*. R Foundation for Statistical Computing Vienna, Austria.

Ross, C. T. & Redhead, D. (2021). DieTryin: An R package for data collection, automated data entry, and post-processing of network-structured economic games, social networks, and other roster-based dyadic data. *Behavior Research Methods*, (pp. 1–21).

Rusch, H. (2014). The evolutionary interplay of intergroup conflict and altruism in humans: a review of parochial altruism theory and prospects for its extension. *Proceedings of the Royal Society B: Biological Sciences*, *281*, 20141539.

Stan Development Team (2020). RStan: The R interface to Stan. R package version 2.21.2.

West, S. A., El Mouden, C. & Gardner, A. (2011). Sixteen common misconceptions about the evolution of cooperation in humans. *Evolution and human behavior*, *32*, 231–262.

Zefferman, M. R. & Mathew, S. (2015). An evolutionary theory of large-scale human warfare: Group-structured cultural selection. *Evolutionary Anthropology: Issues, News, and Reviews*, *24*, 50–61.

Table 1: Model results, Friendship. Coastal site, full set of controls.

Variable	Type	5%CI	Median	95%CI	Location
Age	Alter	-0.71	-0.222	0.29	Coast
Male	Alter	-0.39	0.101	0.61	Coast
Cant Work	Alter	-0.825	-0.312	0.175	Coast
Grip Strength	Alter	-0.115	0.387	0.885	Coast
Depressed	Alter	-0.693	-0.188	0.307	Coast
Food Insecure	Alter	-0.787	-0.279	0.213	Coast
Material Wealth	Alter	0.133	0.657	1.133	Coast
Out Migrated	Alter	-0.85	-0.329	0.15	Coast
Relatedness	Dyadic	1.004	1.505	2.004	Coast
Married	Dyadic	-0.528	0.029	0.472	Coast
Same Sex	Dyadic	1.607	2.074	2.607	Coast
Age	Focal	-0.455	0.054	0.545	Coast
Male	Focal	-0.445	0.046	0.555	Coast
Embera	Focal	-0.474	0.007	0.526	Coast
Cant Work	Focal	-0.461	0.011	0.539	Coast
Grip Strength	Focal	-0.437	0.042	0.563	Coast
Depressed	Focal	-0.467	0.017	0.533	Coast
Food Insecure	Focal	-0.433	0.065	0.567	Coast
Material Wealth	Focal	-0.473	0.029	0.527	Coast
Afrocolombian	Parochial	0.695	1.191	1.695	Coast
Embera	Parochial	1.315	1.807	2.315	Coast
Generalized	Reciprocity	-0.717	-0.017	0.708	Coast
Dyadic	Reciprocity	-0.602	0.296	0.882	Coast

Table 2: Model results, Friendship. Coastal site, no controls.

Variable	Type	5%CI	Median	95%CI	Location
Embera	Focal	-0.45	0.046	0.55	Coast
Afrocolombian	Parochial	0.772	1.24	1.772	Coast
Embera	Parochial	1.332	1.793	2.332	Coast

Table 3: Model results, Friendship. Inland site, full set of controls.

Variable	Type	5%CI	Median	95%CI	Location
Age	Alter	-0.318	0.193	0.682	Inland
Male	Alter	0.209	0.688	1.209	Inland
Cant Work	Alter	-0.718	-0.195	0.282	Inland
Grip Strength	Alter	-0.955	-0.449	0.045	Inland
Depressed	Alter	-0.666	-0.151	0.334	Inland
Food Insecure	Alter	-0.978	-0.484	0.022	Inland
Material Wealth	Alter	-0.413	0.122	0.587	Inland
Out Migrated	Alter	-0.99	-0.462	0.01	Inland
Relatedness	Dyadic	2.002	2.515	3.002	Inland
Married	Dyadic	0.666	1.184	1.666	Inland
Same Sex	Dyadic	1.988	2.471	2.988	Inland
Age	Focal	-0.239	0.255	0.761	Inland
Male	Focal	-0.093	0.393	0.907	Inland
Embera	Focal	-0.968	-0.438	0.032	Inland
Cant Work	Focal	-0.864	-0.364	0.136	Inland
Grip Strength	Focal	-1.104	-0.589	-0.104	Inland
Depressed	Focal	-0.43	0.056	0.57	Inland
Food Insecure	Focal	-0.574	-0.061	0.426	Inland
Material Wealth	Focal	-0.821	-0.314	0.179	Inland
Afrocolombian	Parochial	0.804	1.294	1.804	Inland
Embera	Parochial	1.474	1.97	2.474	Inland
Generalized	Reciprocity	0.639	0.871	0.971	Inland
Dyadic	Reciprocity	-0.28	0.842	0.972	Inland

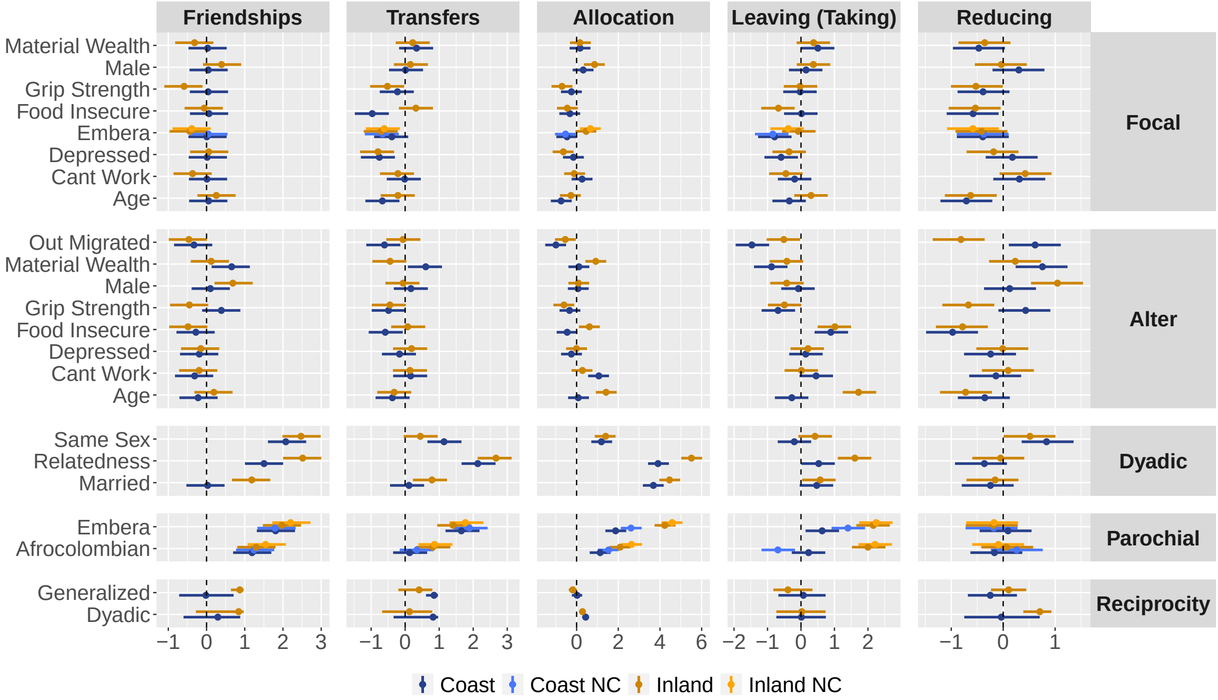

Fig. 2: Multinomial regression results (standardized coefficients) from the Social Relations Model (material wealth on absolute scale). Points and line-ranges show the standardized effects of predictor variables on outcomes (as medians and 90% credible intervals). When the credible intervals exclude the value of zero (plotted as a dashed vertical line), there is evidence of a reliable effect. Each column indicates an independently modeled outcome variable: i) friendship/socializing ties, ii) food/money transfers, iii) coin allocations in the allocation game, iv) coin deductions in the taking game (coded so that positive parameter estimates reflect *leaving* coins), and v) coins paid to reduce alters in the costly reduction game. For each of these outcomes in each community, we fit two models: both included the predictors directly related to parochial altruism (e.g., as in row 4), but the first (NC; *No Controls*) excluded control variables—that is, the predictors in all other rows—and the second included all controls. The key estimates of interest are shown in the "Parochial" row. For example, in the allocation game, both Afrocolombian and Emberá individuals (in both sites) showed a reliably positive tendency to give more to co-ethnics. Likewise, in the taking game, Emberá individuals, as well as inland Afrocolombian individuals, showed a reliably positive tendency to leave more for co-ethnics. However, coastal Afrocolombian individuals showed a reliable tendency to leave more for the ethnic out-group (model with no controls; light blue) or no tendency for preferential out-group exploitation (model with controls; dark blue).

Table 4: Model results, Friendship. Inland site, no controls.

Variable	Type	5%CI	Median	95%CI	Location
Embera	Focal	-0.891	-0.387	0.109	Inland
Afrocolombian	Parochial	1.075	1.544	2.075	Inland
Embera	Parochial	1.721	2.199	2.721	Inland

Table 5: Model results, Food/money transfers. Coastal site, full set of controls.

Variable	Type	5%CI	Median	95%CI	Location
Age	Alter	-0.868	-0.375	0.132	Coast
Male	Alter	-0.333	0.169	0.667	Coast
Cant Work	Alter	-0.353	0.162	0.647	Coast
Grip Strength	Alter	-0.984	-0.487	0.016	Coast
Depressed	Alter	-0.682	-0.166	0.318	Coast
Food Insecure	Alter	-1.07	-0.584	-0.07	Coast
Material Wealth	Alter	0.082	0.605	1.082	Coast
Out Migrated	Alter	-1.142	-0.607	-0.142	Coast
Relatedness	Dyadic	1.657	2.134	2.657	Coast
Married	Dyadic	-0.443	0.112	0.557	Coast
Same Sex	Dyadic	0.655	1.143	1.655	Coast
Age	Focal	-1.168	-0.669	-0.168	Coast
Male	Focal	-0.474	0.011	0.526	Coast
Embera	Focal	-0.911	-0.391	0.089	Coast
Cant Work	Focal	-0.54	-0.013	0.46	Coast
Grip Strength	Focal	-0.746	-0.226	0.254	Coast
Depressed	Focal	-1.301	-0.758	-0.301	Coast
Food Insecure	Focal	-1.479	-0.966	-0.479	Coast
Material Wealth	Focal	-0.177	0.336	0.823	Coast
Afrocolombian	Parochial	-0.354	0.132	0.646	Coast
Embera	Parochial	1.186	1.652	2.186	Coast
Generalized	Reciprocity	0.614	0.85	0.964	Coast
Dyadic	Reciprocity	-0.34	0.821	0.97	Coast

Table 6: Model results, Food/money transfers. Coastal site, no controls.

Variable	Type	5%CI	Median	95%CI	Location
Embera	Focal	-1.186	-0.661	-0.186	Coast
Afrocolombian	Parochial	-0.159	0.343	0.841	Coast
Embera	Parochial	1.423	1.89	2.423	Coast

Table 7: Model results, Food/money transfers. Inland site, full set of controls.

Variable	Type	5%CI	Median	95%CI	Location
Age	Alter	-0.822	-0.321	0.178	Inland
Male	Alter	-0.58	-0.06	0.42	Inland
Cant Work	Alter	-0.359	0.143	0.641	Inland
Grip Strength	Alter	-0.981	-0.447	0.019	Inland
Depressed	Alter	-0.354	0.188	0.646	Inland
Food Insecure	Alter	-0.41	0.076	0.59	Inland
Material Wealth	Alter	-0.964	-0.443	0.036	Inland
Out Migrated	Alter	-0.554	-0.063	0.446	Inland
Relatedness	Dyadic	2.131	2.67	3.131	Inland
Married	Dyadic	0.23	0.788	1.23	Inland
Same Sex	Dyadic	-0.044	0.446	0.956	Inland
Age	Focal	-0.718	-0.211	0.282	Inland
Male	Focal	-0.331	0.155	0.669	Inland
Embera	Focal	-1.218	-0.701	-0.218	Inland
Cant Work	Focal	-0.74	-0.214	0.26	Inland
Grip Strength	Focal	-1.027	-0.52	-0.027	Inland
Depressed	Focal	-1.324	-0.798	-0.324	Inland
Food Insecure	Focal	-0.183	0.316	0.817	Inland
Material Wealth	Focal	-0.276	0.224	0.724	Inland
Afrocolombian	Parochial	0.328	0.812	1.328	Inland
Embera	Parochial	0.942	1.409	1.942	Inland
Generalized	Reciprocity	-0.199	0.411	0.799	Inland
Dyadic	Reciprocity	-0.675	0.129	0.794	Inland

Table 8: Model results, Food/money transfers. Inland site, no controls.

Variable	Type	5%CI	Median	95%CI	Location
Embera	Focal	-1.156	-0.617	-0.156	Inland
Afrocolombian	Parochial	0.391	0.861	1.391	Inland
Embera	Parochial	1.301	1.769	2.301	Inland

Table 9: Model results, Allocation game. Coastal site, full set of controls.

Variable	Type	5%CI	Median	95%CI	Location
Age	Alter	-0.409	0.077	0.591	Coast
Male	Alter	-0.419	0.068	0.581	Coast
Cant Work	Alter	0.554	1.067	1.554	Coast
Grip Strength	Alter	-0.818	-0.339	0.182	Coast
Depressed	Alter	-0.745	-0.252	0.255	Coast
Food Insecure	Alter	-0.956	-0.453	0.044	Coast
Material Wealth	Alter	-0.391	0.108	0.609	Coast
Out Migrated	Alter	-1.505	-0.992	-0.505	Coast
Relatedness	Dyadic	3.428	3.906	4.428	Coast
Married	Dyadic	3.18	3.685	4.18	Coast
Same Sex	Dyadic	0.705	1.189	1.705	Coast
Age	Focal	-1.236	-0.745	-0.236	Coast
Male	Focal	-0.189	0.318	0.811	Coast
Embera	Focal	-0.973	-0.459	0.027	Coast
Cant Work	Focal	-0.237	0.263	0.763	Coast
Grip Strength	Focal	-0.749	-0.248	0.251	Coast
Depressed	Focal	-0.653	-0.153	0.347	Coast
Food Insecure	Focal	-0.829	-0.32	0.171	Coast
Material Wealth	Focal	-0.336	0.166	0.664	Coast
Afrocolombian	Parochial	0.629	1.13	1.629	Coast
Embera	Parochial	1.382	1.872	2.382	Coast
Generalized	Reciprocity	-0.225	0.024	0.267	Coast
Dyadic	Reciprocity	0.314	0.434	0.552	Coast

Table 10: Model results, Allocation game. Coastal site, no controls.

Variable	Type	5%CI	Median	95%CI	Location
Embera	Focal	-1.04	-0.531	-0.04	Coast
Afrocolombian	Parochial	1.019	1.537	2.019	Coast
Embera	Parochial	2.121	2.608	3.121	Coast

Table 11: Model results, Allocation game. Inland site, full set of controls.

Variable	Type	5%CI	Median	95%CI	Location
Age	Alter	0.922	1.412	1.922	Inland
Male	Alter	-0.396	0.084	0.604	Inland
Cant Work	Alter	-0.241	0.28	0.759	Inland
Grip Strength	Alter	-1.115	-0.605	-0.115	Inland
Depressed	Alter	-0.502	-0.007	0.498	Inland
Food Insecure	Alter	0.113	0.615	1.113	Inland
Material Wealth	Alter	0.42	0.919	1.42	Inland
Out Migrated	Alter	-1.038	-0.544	-0.038	Inland
Relatedness	Dyadic	5.023	5.51	6.023	Inland
Married	Dyadic	3.969	4.457	4.969	Inland
Same Sex	Dyadic	0.872	1.395	1.872	Inland
Age	Focal	-0.798	-0.273	0.202	Inland
Male	Focal	0.353	0.862	1.353	Inland
Embera	Focal	-0.052	0.458	0.948	Inland
Cant Work	Focal	-0.597	-0.113	0.403	Inland
Grip Strength	Focal	-1.201	-0.7	-0.201	Inland
Depressed	Focal	-1.147	-0.636	-0.147	Inland
Food Insecure	Focal	-0.94	-0.448	0.06	Inland
Material Wealth	Focal	-0.315	0.157	0.685	Inland
Afrocolombian	Parochial	1.586	2.09	2.586	Inland
Embera	Parochial	3.742	4.23	4.742	Inland
Generalized	Reciprocity	-0.373	-0.184	0.014	Inland
Dyadic	Reciprocity	0.18	0.283	0.386	Inland

Table 12: Model results, Allocation game. Inland site, no controls.

Variable	Type	5%CI	Median	95%CI	Location
Embera	Focal	0.16	0.664	1.16	Inland
Afrocolombian	Parochial	2.142	2.636	3.142	Inland
Embera	Parochial	4.077	4.586	5.077	Inland

Table 13: Model results, Leaving game. Coastal site, full set of controls.

Variable	Type	5%CI	Median	95%CI	Location
Age	Alter	-0.78	-0.274	0.22	Coast
Male	Alter	-0.59	-0.077	0.41	Coast
Cant Work	Alter	-0.044	0.451	0.956	Coast
Grip Strength	Alter	-1.177	-0.684	-0.177	Coast
Depressed	Alter	-0.352	0.142	0.648	Coast
Food Insecure	Alter	0.405	0.891	1.405	Coast
Material Wealth	Alter	-1.403	-0.879	-0.403	Coast
Out Migrated	Alter	-1.952	-1.467	-0.952	Coast
Relatedness	Dyadic	0.01	0.529	1.01	Coast
Married	Dyadic	-0.039	0.471	0.961	Coast
Same Sex	Dyadic	-0.696	-0.2	0.304	Coast
Age	Focal	-0.855	-0.348	0.145	Coast
Male	Focal	-0.36	0.152	0.64	Coast
Embera	Focal	-1.281	-0.788	-0.281	Coast
Cant Work	Focal	-0.69	-0.189	0.31	Coast
Grip Strength	Focal	-0.531	-0.025	0.469	Coast
Depressed	Focal	-1.091	-0.594	-0.091	Coast
Food Insecure	Focal	-0.506	0.016	0.494	Coast
Material Wealth	Focal	-0.002	0.505	0.998	Coast
Afrocolombian	Parochial	-0.275	0.226	0.725	Coast
Embera	Parochial	0.137	0.632	1.137	Coast
Generalized	Reciprocity	-0.676	0.071	0.735	Coast
Dyadic	Reciprocity	-0.737	0.012	0.743	Coast

Table 14: Model results, Leaving game. Coastal site, no controls.

Variable	Type	5%CI	Median	95%CI	Location
Embera	Focal	-1.375	-0.842	-0.375	Coast
Afrocolombian	Parochial	-1.179	-0.69	-0.179	Coast
Embera	Parochial	0.912	1.402	1.912	Coast

Table 15: Model results, Leaving game. Inland site, full set of controls.

Variable	Type	5%CI	Median	95%CI	Location
Age	Alter	1.246	1.719	2.246	Inland
Male	Alter	-0.919	-0.428	0.081	Inland
Cant Work	Alter	-0.492	0.012	0.508	Inland
Grip Strength	Alter	-0.989	-0.494	0.011	Inland
Depressed	Alter	-0.314	0.211	0.686	Inland
Food Insecure	Alter	0.497	1.012	1.497	Inland
Material Wealth	Alter	-0.935	-0.422	0.065	Inland
Out Migrated	Alter	-1.025	-0.512	-0.025	Inland
Relatedness	Dyadic	1.101	1.609	2.101	Inland
Married	Dyadic	0.034	0.573	1.034	Inland
Same Sex	Dyadic	-0.078	0.42	0.922	Inland
Age	Focal	-0.2	0.301	0.8	Inland
Male	Focal	-0.125	0.372	0.875	Inland
Embera	Focal	-0.565	-0.08	0.435	Inland
Cant Work	Focal	-0.952	-0.452	0.048	Inland
Grip Strength	Focal	-0.513	-0.027	0.487	Inland
Depressed	Focal	-0.854	-0.355	0.146	Inland
Food Insecure	Focal	-1.189	-0.676	-0.189	Inland
Material Wealth	Focal	-0.13	0.378	0.87	Inland
Afrocolombian	Parochial	1.524	1.998	2.524	Inland
Embera	Parochial	1.654	2.16	2.654	Inland
Generalized	Reciprocity	-0.826	-0.39	0.342	Inland
Dyadic	Reciprocity	-0.736	0.024	0.735	Inland

Table 16: Model results, Leaving game. Inland site, no controls.

Variable	Type	5%CI	Median	95%CI	Location
Embera	Focal	-0.918	-0.379	0.082	Inland
Afrocolombian	Parochial	1.716	2.211	2.716	Inland
Embera	Parochial	1.736	2.247	2.736	Inland

Table 17: Model results, Reducing game. Coastal site, full set of controls.

**Variable**	**Type**	**5%CI**	**Median**	**95%CI**	**Location**
Age	Alter	-0.873	-0.354	0.127	Coast
Male	Alter	-0.368	0.125	0.632	Coast
Cant Work	Alter	-0.654	-0.14	0.346	Coast
Grip Strength	Alter	-0.091	0.432	0.909	Coast
Depressed	Alter	-0.753	-0.241	0.247	Coast
Food Insecure	Alter	-1.484	-0.973	-0.484	Coast
Material Wealth	Alter	0.239	0.756	1.239	Coast
Out Migrated	Alter	0.109	0.615	1.109	Coast
Relatedness	Dyadic	-0.928	-0.362	0.072	Coast
Married	Dyadic	-0.798	-0.245	0.202	Coast
Same Sex	Dyadic	0.355	0.835	1.355	Coast
Age	Focal	-1.208	-0.709	-0.208	Coast
Male	Focal	-0.205	0.302	0.795	Coast
Embera	Focal	-0.892	-0.392	0.108	Coast
Cant Work	Focal	-0.19	0.313	0.81	Coast
Grip Strength	Focal	-0.878	-0.387	0.122	Coast
Depressed	Focal	-0.336	0.177	0.664	Coast
Food Insecure	Focal	-1.088	-0.579	-0.088	Coast
Material Wealth	Focal	-0.967	-0.47	0.033	Coast
Afrocolombian	Parochial	-0.631	-0.168	0.369	Coast
Embera	Parochial	-0.454	0.094	0.546	Coast
Generalized	Reciprocity	-0.68	-0.248	0.265	Coast
Dyadic	Reciprocity	-0.75	-0.035	0.704	Coast

Table 18: Model results, Reducing game. Coastal site, no controls.

**Variable**	**Type**	**5%CI**	**Median**	**95%CI**	**Location**
Embera	Focal	-0.894	-0.374	0.106	Coast
Afrocolombian	Parochial	-0.238	0.261	0.762	Coast
Embera	Parochial	-0.724	-0.182	0.276	Coast

Table 19: Model results, Reducing game. Inland site, full set of controls.

**Variable**	**Type**	**5%CI**	**Median**	**95%CI**	**Location**
Age	Alter	-1.216	-0.724	-0.216	Inland
Male	Alter	0.537	1.046	1.537	Inland
Cant Work	Alter	-0.409	0.098	0.591	Inland
Grip Strength	Alter	-1.171	-0.669	-0.171	Inland
Depressed	Alter	-0.516	-0.01	0.484	Inland
Food Insecure	Alter	-1.295	-0.782	-0.295	Inland
Material Wealth	Alter	-0.272	0.233	0.728	Inland
Out Migrated	Alter	-1.357	-0.814	-0.357	Inland
Relatedness	Dyadic	-0.594	-0.049	0.406	Inland
Married	Dyadic	-0.707	-0.155	0.293	Inland
Same Sex	Dyadic	0.007	0.516	1.007	Inland
Age	Focal	-1.124	-0.627	-0.124	Inland
Male	Focal	-0.546	-0.036	0.454	Inland
Embera	Focal	-0.919	-0.416	0.081	Inland
Cant Work	Focal	-0.069	0.424	0.931	Inland
Grip Strength	Focal	-1.006	-0.525	-0.006	Inland
Depressed	Focal	-0.705	-0.184	0.295	Inland
Food Insecure	Focal	-1.05	-0.535	-0.05	Inland
Material Wealth	Focal	-0.86	-0.355	0.14	Inland
Afrocolombian	Parochial	-0.422	0.062	0.578	Inland
Embera	Parochial	-0.72	-0.189	0.28	Inland
Generalized	Reciprocity	-0.234	0.106	0.444	Inland
Dyadic	Reciprocity	0.388	0.707	0.929	Inland

Table 20: Model results, Reducing game. Inland site, no controls.

Variable	Type	5%CI	Median	95%CI	Location
Embera	Focal	-1.082	-0.583	-0.082	Inland
Afrocolombian	Parochial	-0.596	-0.092	0.404	Inland
Embera	Parochial	-0.711	-0.175	0.289	Inland

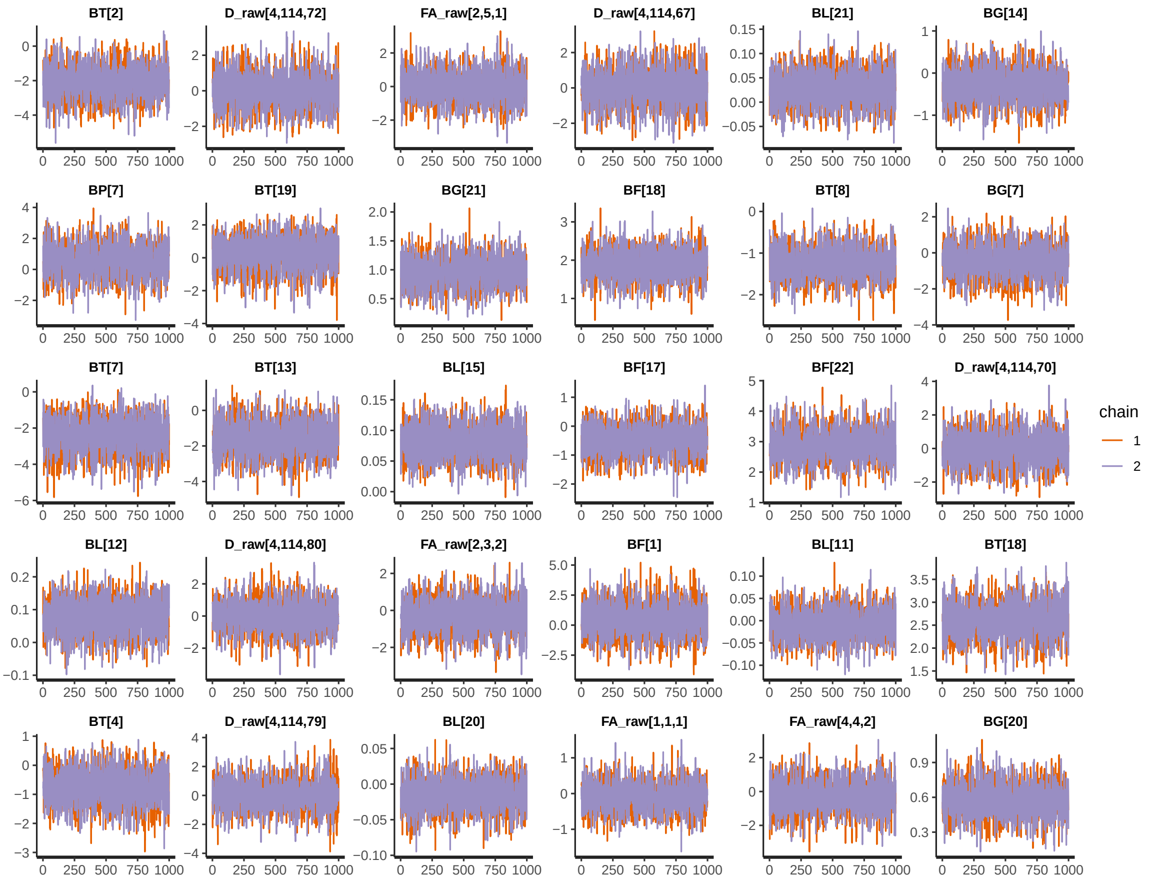

Fig. 3: Model 1: Coastal, all covariates. Traceplots of a sample of parameters from each model. All chains show good mixing, and convergence to the same posterior region.

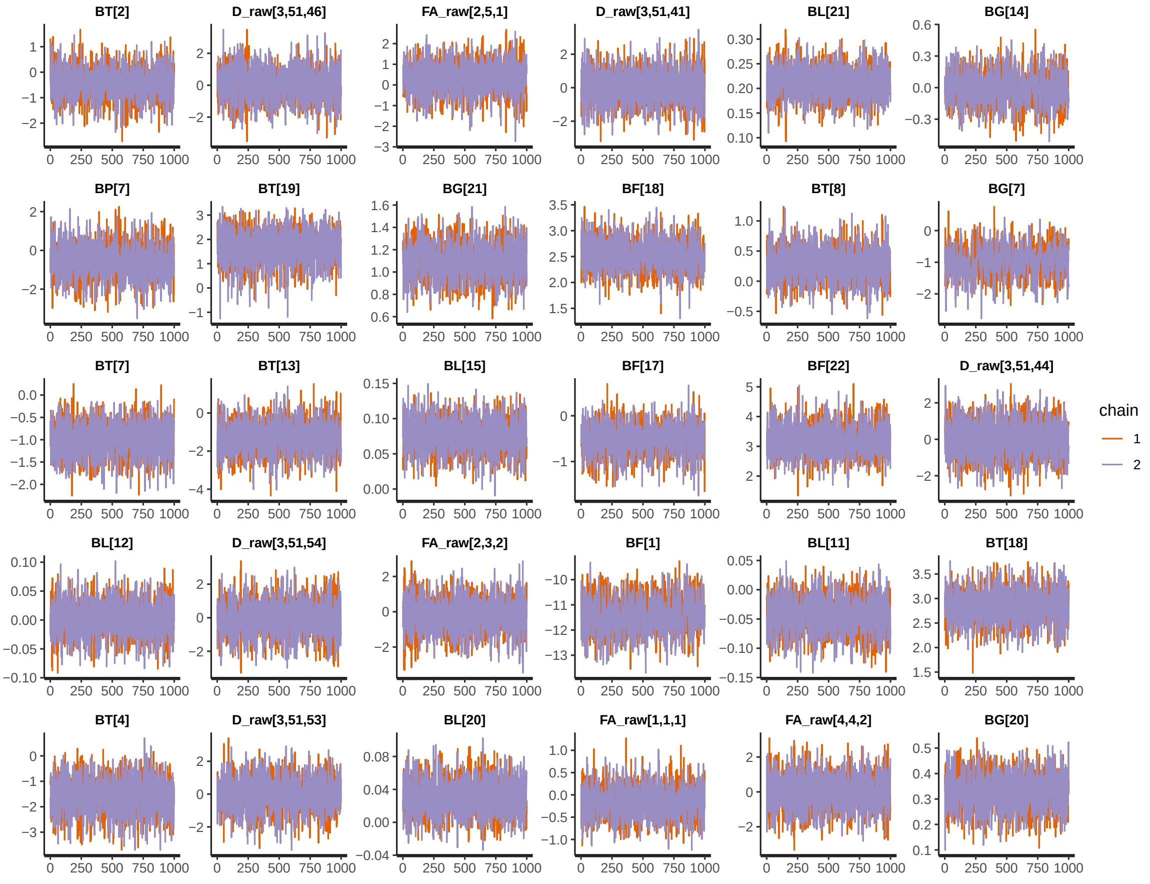

Fig. 4: Model 2: Inland, all covariates. Traceplots of a sample of parameters from each model. All chains show good mixing, and convergence to the same posterior region.

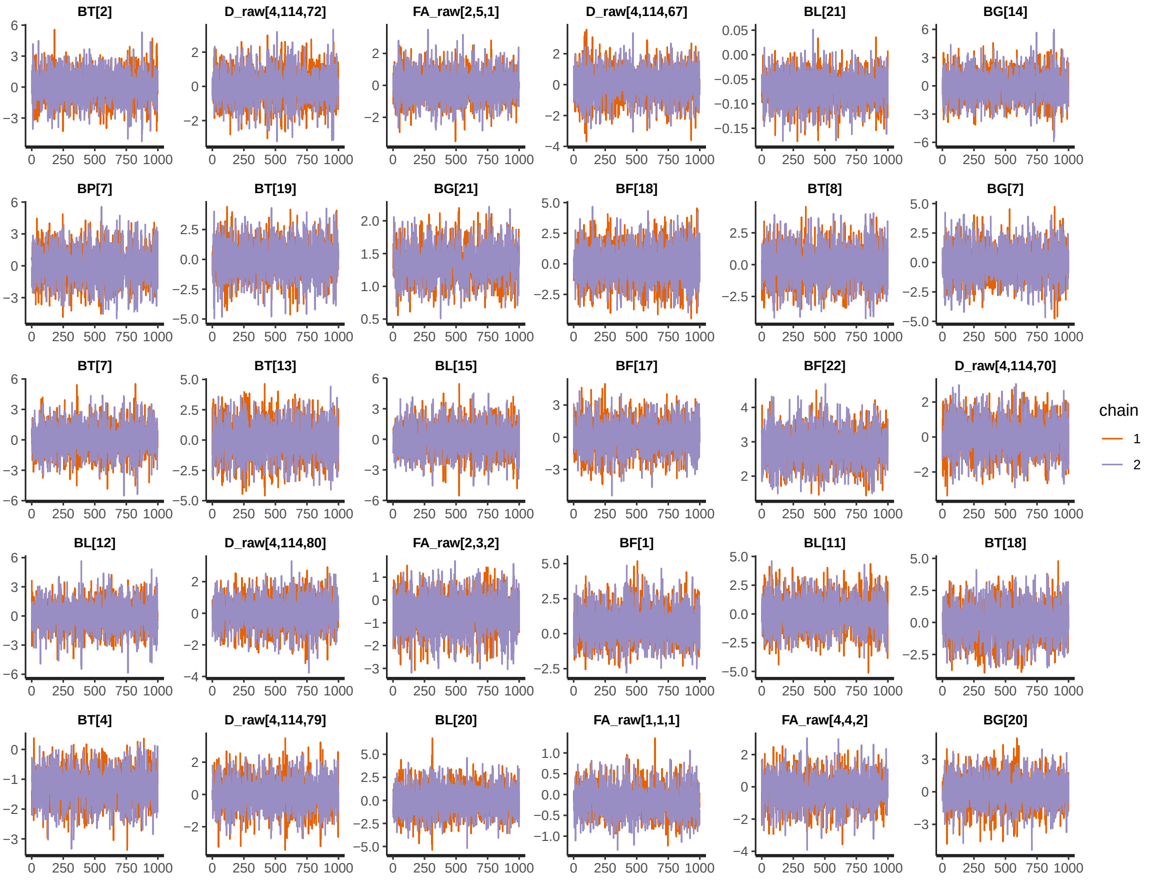

Fig. 5: Model 3: Coastal, only ethnicity. Traceplots of a sample of parameters from each model. All chains show good mixing, and convergence to the same posterior region.

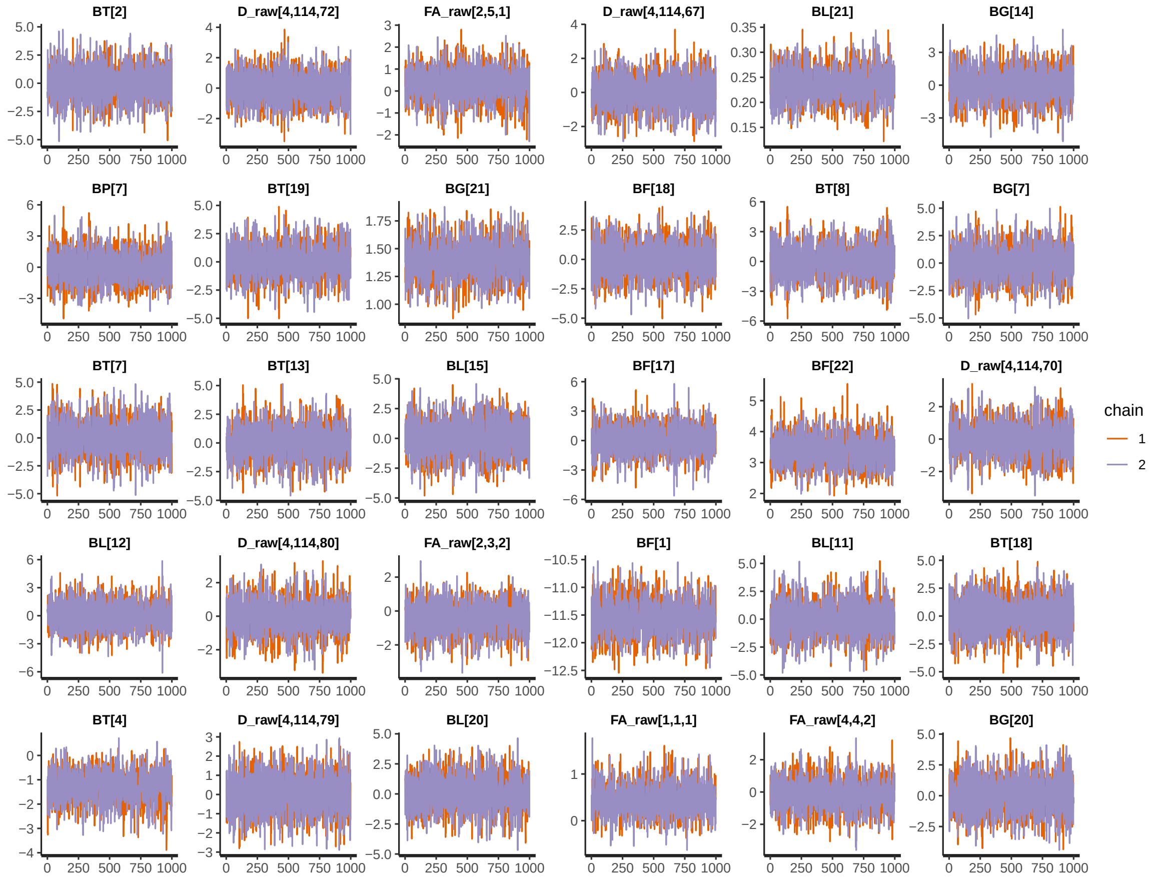

Fig. 6: Model 4: Inland, only ethnicity. Traceplots of a sample of parameters from each model. All chains show good mixing, and convergence to the same posterior region.
